# Heparin binding preference and structures in the fibroblast growth factor family parallel their evolutionary diversification

**DOI:** 10.1098/rsob.150275

**Published:** 2016-03-30

**Authors:** Yong Li, Changye Sun, Edwin A. Yates, Chao Jiang, Mark C. Wilkinson, David G. Fernig

**Affiliations:** 1 Department of Biochemistry, Institute of Integrative Biology, University of Liverpool, Biosciences Building, Crown Street, Liverpool L69 7ZB, UK; 2 School of Pharmaceutical Science, Wenzhou Medical University, Chashan University Park, Wenzhou 325035, China

**Keywords:** fibroblast growth factor, glycosaminoglycan, heparan sulfate, specificity, molecular recognition, heparin binding site

## Abstract

The interaction of a large number of extracellular proteins with heparan sulfate (HS) regulates their transport and effector functions, but the degree of molecular specificity underlying protein–polysaccharide binding is still debated. The 15 paracrine fibroblast growth factors (FGFs) are one of the paradigms for this interaction. Here, we measure the binding preferences of six FGFs (FGF3, FGF4, FGF6, FGF10, FGF17, FGF20) for a library of modified heparins, representing structures in HS, and model glycosaminoglycans, using differential scanning fluorimetry. This is complemented by the identification of the lysine residues in the primary and secondary binding sites of the FGFs by a selective labelling approach. Pooling these data with previous sets provides good coverage of the FGF phylogenetic tree, deduced from amino acid sequence alignment. This demonstrates that the selectivity of the FGFs for binding structures in sulfated polysaccharides and the pattern of secondary binding sites on the surface of FGFs follow the phylogenetic relationship of the FGFs, and so are likely to be the result of the natural selection pressures that led to the expansion of the FGF family in the course of the evolution of more complex animal body plans.

## 1. Introduction

The glycosaminoglycan heparan sulfate (HS) regulates many aspects of cell communication by means of binding to over 435 extracellular proteins and thereby controlling their activities [1] (reviewed in [2–4]). Two classic examples are the activation of antithrombin III by the polysaccharide, which contributes to the regulation of coagulation [5], and the control of the transport and effector functions of the paracrine fibroblast growth factors (FGFs) by their binding to HS [6–10]. A major challenge is to understand the structural basis of the interactions of proteins with HS and to what extent any molecular specificity and selectivity of these interactions is of functional significance.

HS consists of repeating disaccharide units joined by 1–4 linkages. The HS chains are always synthesized attached to a core protein to form HS proteoglycans. It is the core protein that directs the HS chains to their functional location, which can be the cell surface or the extracellular matrix. Heparin, often used as an experimental proxy for HS on account of its underlying structural similarity, is nevertheless a more sulfated structure. The repeating units of HS consist of a glucuronic acid (GlcA) or its C5 epimer iduronic acid (IdoA) and d-glucosamine (GlcN). The glucosamine may be *N*-acetylated (GlcNAc), *N*-sulfated (GlcNS) or unsubstituted (GlcN). The biosynthetic pathway has been proposed to have two branches [11]: the major branch is the chain modified by the *N*-deacetylase/*N*-sulfotransferases to replace the *N*-acetyl group of glucosamine with an *N*-sulfate group [12,13] and may be followed by C5 epimerization with C5 epimerase [14] and *O*-sulfation with 2-*O*, 6-*O* and 3-*O* sulfotransferases [15,16]; the minor branch arises from the position in the scheme at which that the HS epimerase applies on the chain at an early point, which converts the GlcA–GlcNAc to IdoA–GlcNAc. Because almost all the other modifications depend on the presence of *N*-sulfated glucosamine, the result is that HS chains have a domain structure: NA domains with no sulfation structure, NS domains of highly sulfated structures and NA/NS domains comprising mixed disaccharides of GlcNAc and GlcNS [17] (reviewed in [12,18]). The sulfated structures are considered to be of functional significance, forming the protein binding domains [2].

The FGF family of 22 proteins has been divided into seven subfamilies by phylogenetic analysis [19]. Based on their mechanisms of action, FGFs can be classified into three types: intracrine, paracrine and endocrine. Only the paracrine FGFs bind to HS. Evidence for control by HS of FGF transport comes from a variety of experimental systems. For example, mutations in the gene encoding sugarless (sgl) and sulfateless (sfl), which are part of the *Drosophila* HS chain biosynthetic machinery, were identified as producing similar phenotypes to Wingless (Wg) or Hedgehog (Hh) signalling mutants [13]. Interactions with HS occurring in the extracellular matrix have been shown directly to regulate the diffusion of FGFs [8,20] and so can determine the shape of FGF concentration gradients in development [21,22], as well as the storage and release of FGFs in tissue homeostasis [9,10]. The growth factor/morphogen-type signals generated by FGFs require the assembly of the ternary complex of FGF ligand, FGF receptor (FGFR) and HS, which engages both the ligand and receptor [6,7]. Thus, in this respect, HS acts as a co-receptor.

In terms of the specificity of interactions of proteins with HS, there are different paradigms and views. One paradigm is the activation of antithrombin III by its binding to a specific pentasaccharide sequence in heparin [23], which has been successfully transformed into a synthetic anticoagulant, Arixtra [24,25]. It was the higher affinity saccharide unit salt-eluted from an antithrombin III affinity column that was originally identified, but it was not the only sequence that bound antithrombin III and was able to activate it. More recent findings are that activity relates to thermal stabilization of antithrombin III [26], and many oligosaccharide structures have now been shown both to possess a high affinity for antithrombin III and to exert strong anticoagulant activity [27,28]. With other proteins, there is even less consensus. Thus, with FGFs, highly specific binding structures in heparin and in HS have been sought [29]. In other experiments, however, the conclusion was that the charge density of the polysaccharide was the major determinant of binding selectivity (reviewed in [30]).

In a recent attempt to understand the extent, if any, of selectivity of FGFs for binding to HS, the molecular basis of the interactions between six FGFs from five subfamilies and HS was characterized in depth. The results suggested that there is a degree of selectivity in FGF–heparin interactions, and this reflects the evolution of the FGF family members [31], which parallels the specificity of FGF ligands for FGFRs [32]. However, this work is limited in its coverage: two FGFs from one subfamily and one from each of four other subfamilies. Therefore, alternative explanations are quite possible. Consequently, here we characterize the interactions with HS of a further six FGFs from two perspectives. The preference of FGFs for a particular sugar structure has been determined using differential scanning fluorimetry (DSF) and a library of chemically modified heparins, heparin-derived oligosaccharides and model glycosaminoglycan. A protect and label approach is then used to identify lysine side chains involved in heparin binding and so map the primary and secondary HS binding sites in the FGFs. Pooling the present data with those acquired previously [31,33,34] demonstrates that the FGFs show clear selectivity for binding structures and that this, along with the pattern of secondary binding sites on the surface of the FGFs, follows the phylogeny established by amino acid sequence alignment. Thus, the molecular basis of the interactions of FGFs with HS and their preference for particular isoforms of the FGFR have followed the expansion and specialization of the FGF family that occurred during the course of the evolution of the more complex body plans of animals.

## 2. Material and methods

### 2.1. Materials

Heparin (17 kDa average molecular mass, Celsus Lab, Cincinnati, OH) was used in all assays, and as the starting material for the production of modified derivatives and oligosaccharides. Different chemically modified heparin derivatives D1–9 (table 1; [35]), and cationic forms were produced, as described [36], whereas oligosaccharides with degrees of polymerization (dp) dp2–dp12 were obtained from Iduron (Manchester, UK). Porcine mucosal HS, hyaluronic acid (HA, not sulfated) and chondroitin sulfate C (CS-C, average sulfate per disaccharide unit, 1) were from Sigma (Gillingham, Dorset, UK); Dermatan sulfate (DS, average sulfate per disaccharide unit, 1) were from Iduron.

**Table 1.** Nomenclature and structures of chemically modified heparin structures. I stands for iduronate, and A stands for the amino sugar glucosamine. ^a^Numbers refer to the ring position of carbon atoms. The average number of sulfate groups per disaccharide is also indicated.

analogue	predominant repeat	IdoUA-2	GlcN-6	GlcN-2	IdoUA-3	GlcN-3^a^	sulfate groups per disaccharide
D1 (heparin)	I_2S_A^6S^Ns	SO_3_^−^	SO_3_^−^	SO_3_^−^	OH	OH	2.4
D2	I_2S_A^6S^NAc	SO_3_^−^	SO_3_^−^	COCH_3_	OH	OH	1.8
D3	I_2OH_A^6S^Ns	OH	SO_3_^−^	SO_3_^−^	OH	OH	1.9
D4	I_2S_A^6S^Ns	SO_3_^−^	OH	SO_3_^−^	OH	OH	1.8
D5	I_2OH_A^6S^NAc	OH	SO_3_^−^	COCH_3_	OH	OH	1.2
D6	I_2S_A^6OH^NAc	SO_3_^−^	OH	COCH_3_	OH	OH	0.8
D7	I_2OH_A^6OH^Ns	OH	OH	SO_3_^−^	OH	OH	0.8
D8	I_2OH_A^6OH^NAc	OH	OH	COCH_3_	OH	OH	0
D9	I_2S,3S_A^6S^_3S_Ns	SO_3_^−^	SO_3_^−^	SO_3_^−^	SO_3_^−^	SO_3_^−^	4.4

### 2.2. Recombinant human fibroblast growth factors

cDNA encoding Histag-FGF4 (UniProt accession number P08620; residues 31–206) was transformed into C41 (DE3) cells and expressed by inducing with 1 mM isopropyl 1-thio-β-d-galactopyranoside at 37°C for 3 h. After cell lysis by sonication and clarification by centrifugation at 38 000*g* for 30 min, the supernatant was loaded onto a 1 ml affinity HiTrap heparin HP column (GE Healthcare, Amersham, Bucks, UK), washed with buffer (50 mM Tris–Cl, 0.3 M NaCl, pH 7.2, 30 ml) and then eluted with 1 M NaCl in 50 mM Tris buffer. FGF4 required further purification with a cation-exchange HiTrap 1 ml SP HP column to remove the contaminants, as described previously for FGF2 [33]. HaloTag (HT)-FGF17 and HT-FGF6 were expressed and purified as described [37]; the HT-FGF17 protein was digested overnight by mixing with TEV protease at ratio 40 : 1. The sample was then loaded on a HiTrap Q column. FGF17 eluted in the flow through fraction, because it did not bind to this anion-exchange matrix, whereas the anionic HaloTag protein bound to the column. The FGF17 was then further purified by affinity chromatography on a 1 ml Hitrap heparin column. Histag-FGF3, HT-FGF6, Histag-FGF10 and Histag-FGF20 were expressed and purified, as described [37]. Because proteins were produced in *Escherichia coli*, they will not be glycosylated.

### 2.3. Size exclusion chromatography–multi-angle laser light scattering

Analysis of the solution molecular mass was performed by separation of proteins on 25 ml Superdex 200 HR10/300 columns (GE Healthcare) connected in series with a Wyatt Dawn8+ and Wyatt Optilab T-rEX (Wyatt Technology, Haverhill, UK) at 22°C. Samples (100 µl, 100 µg protein) were filtered and then applied to the column, which was developed in 150 mM NaCl buffered with either 50 mM HEPES or 50 mM Tris–Cl, both pH 7.4 at a flow rate of 0.75 ml min^−1^.

### 2.4. Differential scanning fluorimetry

DSF was performed with a 7500 fast real-time PCR (RT-PCR) instruction (software version 1.4.0, Applied Biosystems, Paisley, UK), as described [31,33]. The different sugars (100 µM, 3.5 µl in HPLC grade water) and FGFs (50 µM, 3.5 µl) as 10-fold concentrated stock solutions, phosphate-buffered saline (PBS: NaCl 137 mM, KCl 2.7 mM, Na_2_HPO_4_ 10 mM, KH_2_PO_4_ 1.8 mM; 24.5 µl), and freshly prepared 100-fold stock solution Sypro Orange dye (3.5 µl; Life Technologies, Paisley UK) were added to a Fast Optical 96 Well Reaction plate (Life Technologies) kept on ice. After sealing with Optical Adhesive Film (Life Technologies), the plate was directly analysed in the RT-PCR instrument with a heating cycle covering a gradient between 32 and 81°C in 99 steps of 20 s. First derivatives of the melting curves were calculated with Origin 7 (OriginLab Corp., Northampton, UK). For each sugar, at least two experiments each in triplicate was performed and analysed. The mean melting temperature *T*_m_ and the standard error (s.e.) were calculated based on the six repeats. Data were normalized as: [*T*_m_ *x* − *T*_m_ PBS]/[*T*_m_ hep − *T*_m_ PBS], where *T*_m_ *x* is the *T*_m_ of protein in the presence of the heparin derivative; *T*_m_ PBS is the *T*_m_ of the protein in PBS, and *T*_m_ hep is the *T*_m_ of the protein in the presence of heparin. The relative stability of protein in PBS buffer was set to 0, whereas the relative stability of the protein in the presence of heparin was set to 1.

### 2.5. Protect and label identification of lysines involved in heparin binding (structural proteomics)

#### 2.5.1. Lysine protection

The identification of lysines in heparin binding sites (HBS) was according to Ori *et al*. [34] with minor modifications. A heparin minicolumn was made by placing a plastic air filter at the end of a small pipette tip into which 30 µl of AF-heparin beads (Tosoh Biosciences, Stuttgart, Germany; binding capacity 4 mg antithrombin III ml^−1^ resin) was packed. A 5 ml syringe was used to pack the minicolumn and dispense buffer. The heparin column was equilibrated four times with 50 µl of PB 150 buffer (17.9 mM Na_2_HPO_4_, 2.1 mM NaH_2_PO_4_, 150 mM NaCl, pH 7.8). A minimum of 40 µg FGF protein was loaded onto the heparin column, and the loading was repeated three times with the same sample. After binding, the column was washed with PB 150 buffer four times. To acetylate exposed lysines, the minicolumn was then quickly rinsed with 20 µl of PB 150 containing 50 mM sulfo-NHS–acetate (Life Technologies, Paisley, UK) and then incubated for 5 min with 20 µl of fresh PB 150 containing 50 mM sulfo-NHS–acetate at room temperature. After acetylation, the minicolumn was washed with 50 µl of PB 150 buffer, and acetylated protein was eluted from heparin with 2 × 20 µl elution buffer (45 mM Na_2_HPO_4_, 5 mM NaH_2_PO_4_, 2 M NaCl, pH 7.8).

#### 2.5.2. Heparin binding site lysine biotinylation

Acetylated protein was diluted with 200 µl of PB buffer and concentrated with a 5 kDa MWCO centrifugal filter (Sartorius, Epsom, UK) by centrifugation for 15 min at 11 200*g*. The volume was adjusted to 37.2 µl with PB buffer, and any remaining amino groups were biotinylated by the addition of 2.8 µl 145 mM NHS–biotin (Life Technologies) in dimethylsulfoxide and 30 min incubation at room temperature. The biotinylation reaction was quenched with 4 µl of 1 M Tris, pH 7.5. Then, the sample was transferred to a desalting centrifugal column (7 kDa MWCO, Thermo Scientific, Rockford, UK), covered with 70 µl HPLC grade water and centrifuged for 2 min. Samples were frozen at −80°C for 10 min and dried by centrifugal evaporation.

#### 2.5.3. Protein digestion

Dried sample was dissolved with 25 µl 8 M urea, 400 mM NH_4_HCO_3_, pH 7.8 and 2.5 µl 45 mM DTT and incubated for 15 min at 56°C. Then, the samples were carbamidomethylated with 2.5 µl of freshly made 0.1 M iodoacetamide for 15 min at room temperature in the dark. Proteins were diluted with 70 µl HPLC grade water and digested overnight with 1 µg MS-grade protease (trypsin, chymotrypsin, thermolysin or Glu-C; Promega, Southampton, UK).

#### 2.5.4. Identification of labelled peptides

Biotinylated/acetylated peptides were made up to 0.5% (w/v) trifluoroacetic acid (TFA) and desalted using C18 Zip Tips (Millipore). The latter were pre-wetted with 100% (v/v) acetonitrile (ACN) and then pre-equilibrated with 0.1% (w/v) TFA in water. The peptides were loaded on the Zip Tip and then washed with 10 µl 0.1% (w/v) TFA. Finally, the peptides were eluted with two aliquots of 4–6 µl 50% (v/v) ACN. The samples were concentrated by rotary evaporation. Analyses were performed on a MALDI-TOF mass spectrometer (Waters, Manchester, UK). The MS spectra were produced by MassLynx v.4.0 and then analysed with the MS-digest tool of the Protein Prospector package v. 5.12.4 with the following parameters: considered modification, acetyl (K), biotin (K), carbamidomethyl (C), carboxymethyl (C); protease used, trypsin/chymotrypsin, thermolysin or Glu-C; missed cleavages, 5; minimum–maximum mass: 800–4000.

## 3. Results

We have used two approaches and multiple representatives of the different human heparin binding FGF subfamilies, FGF3 and 10 (FGF7 subfamily), FGF16 and 20 (FGF9 subfamily), FGF4 and 6 (FGF4 subfamily), and FGF8 and 17 (FGF8 subfamily) to gain an insight into the selectivity and structural basis of the interaction of FGFs with glycosaminoglycan. DSF makes use of an environment sensitive dye (Sypro Orange), which when bound to aromatic residues produces a high fluorescence; these residues are exposed when proteins are thermally denatured [33]. This allows measurement of the extent of stabilization of the structure of FGFs that occurs upon binding different glycosaminoglycan structures. The protect and label approach identifies lysine residues that are engaged in direct interactions with heparin and so determines the likely binding sites of the polysaccharide in the FGFs. It is capable of identifying lysine residues in both the primary, higher affinity canonical binding site, and in the much lower affinity secondary binding sites [31,34].

### 3.1. Thermal stabilization of fibroblast growth factors by interaction with heparin

**Figure 1.**
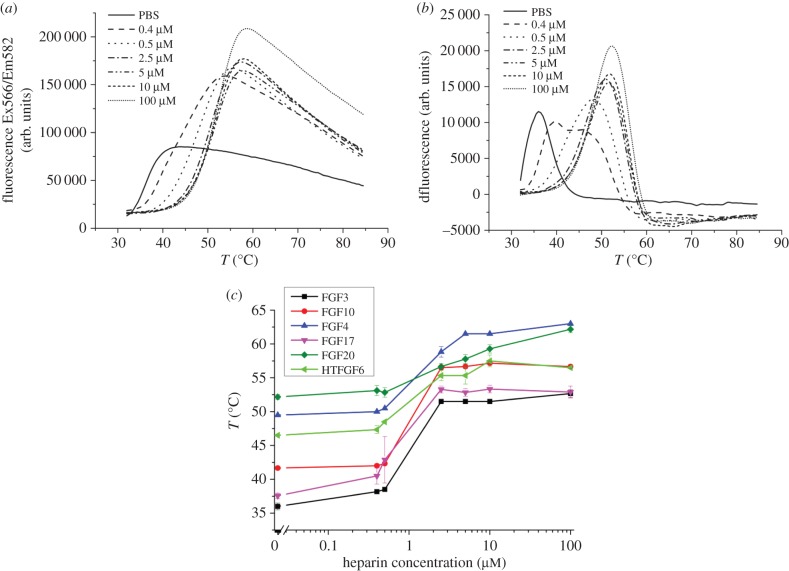
Stabilization effect of heparin on FGF3. Differential scanning fluorimetry of 5 µM FGF3 in the presence of varying concentrations of heparin. (*a*) Melting curve profiles of FGF3 (5 µM) with a range of heparin concentrations (0–100 µM). (*b*) First derivative of the melting curves of FGF3 in (*a*). (*c*) Heparin-dependence of the melting temperature (*T*_m_) of FGF3, FGF10, FGF4, FGF17, FGF20 and HT-FGF6, *T*_m_ is the mean of triplicates ± s.e.

The change in fluorescence was measured as temperature was increased with 5 µM of each FGF, and then the first derivative was calculated to determine the melting temperature. Only one melting curve and corresponding derivative per sample is shown in the figures for clarity. Complete datasets are shown in the electronic supplementary material. In the case of FGF3, as the concentration of heparin increased, the melting curves were displaced to the right, indicating that the FGF3 melting temperature increased (figure 1*a*). It should be noted that there are two competing processes, the binding of the dye Sypro orange to exposed aromatic residues, which causes an increase in fluorescence as the FGF3 unfolds, and the aggregation of the unfolded FGF3, which will re-bury these side chains and cause a decrease in fluorescence [33]. The amplitude of the change in fluorescence thus depends on the total concentration of protein and its aggregation. It is clear that in the presence of heparin the unfolded FGF3 aggregates less, because the amplitude of fluorescence is higher (figure 1*a*). The first derivative of the melting curves identifies the melting temperature (figure 1*b*), which can then be plotted as a function of heparin concentration (figure 1*c*). Collectively, these experiments show that heparin has a concentration-dependent effect on the thermal stability of all the FGFs tested, because their melting temperature progressively increased as the concentration of heparin increased (figure 1*c*). The melting temperatures (*T*_m_) of FGF3 and FGF17 are 36°C and 37.5°C, respectively, whereas the *T*_m_ of FGF4 (49.5°C), FGF10 (41.6°C) and FGF20 (52.2°C) [38,39] are considerably higher (figure 1*c*). In the case of FGF6, the protein aggregates when the *N*-terminal HaloTag fusion protein is removed [37], so the DSF assay was performed on the fusion protein. Two distinct peaks are observed, one at 46.5°C and the other at 58.5°C (electronic supplementary material, figure S3*b,c*). The lower melting temperature (46.5°C) is assigned to FGF6 for two reasons. First, the *T*_m_ of purified HaloTag corresponds to the second peak (electronic supplementary material, figure S3*b*), whereas the peak at 46.5°C is shifted to higher temperature when the protein is incubated with heparin (figure 1*c*) and only the FGF6 moiety binds the polysaccharide [37]. Thus, not only are FGF4, FGF6, FGF10 and FGF20 more stable than FGF3 and FGF17 in the absence of heparin, but also, interestingly, human FGF3 and FGF17 would under these conditions be unstable at normal body temperature (figure 1*c*).

To determine the effects of binding heparin, a range of heparin concentrations (0–100 µM) were tested against a fixed concentration (5 µM) of FGFs, and the melting temperature was calculated at each concentration of polysaccharide. The melting curves show that a stabilizing effect of heparin on FGF3 and FGF10 is apparent from 0.5 (heparin–FGF molar ratio, 1 : 10) to 2.5 µM heparin (heparin–FGF molar ratio, 1 : 2) and is then unchanged at higher concentrations of heparin (figure 1*c* and electronic supplementary material, S1*c*). The melting temperature of FGF6 was increased by heparin. Although the signal from the more stable HaloTag overlapped in part that from FGF6 when the latter was fully stabilized by heparin, the point of inflection associated with the increase in fluorescence arising from the unfolding of the FGF6 moiety of the fusion protein remained distinct (electronic supplementary material, figure S3*b*). Therefore, this approach could be used to measure the relative stabilizing effect of the interaction of FGF6 with glycosaminoglycans. The effect of heparin on the melting temperature of FGF4, FGF6 and FGF17 is similar, with stabilization becoming apparent at 0.5 µM heparin (heparin–FGF, 10 : 1) and reaching a maximum around 2.5 µM heparin (heparin–FGF, 1 : 2). However, the stabilizing effect of heparin on FGF20, which is apparent at 0.5 µM heparin, does not reach a maximum even with 100 µM heparin, and so is distinct from the other four FGFs. The thermal stabilization of FGF20 by heparin was the lowest at 10°C, whereas for FGF4, FGF6, FGF10 and FGF17 it ranged from 11 to 15°C and was 17°C for FGF3. Thus, in the case of FGF3 and FGF17, binding to heparin raises their melting temperature well above body temperature.

### 3.2. Analysis of sugar binding selectivity by differential scanning fluorimetry

The structures in the polysaccharide required for binding these FGFs were then determined by measuring the stabilization effect of a library of model glycosaminoglycans and their derivatives. The molar ratio of FGF–polysaccharide used in this experiment was approximately 1 : 2 (FGF, 5 µM; polysaccharide, 10 µM).

**Figure 2.**
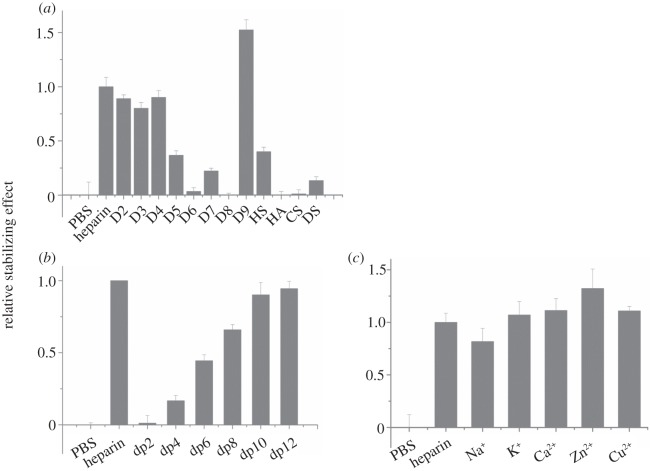
Differential scanning fluorimetry (DSF) analysis of binding of glycosaminoglycan derivatives to FGF3. DSF of 5 µM FGF3 was performed in the presence of a range of heparin-based poly- and oligosaccharides (all 10 µM) and the thermal stabilization relative to the PBS control (=0) and heparin (=1) was calculated (see ‘Differential scanning fluorimetry’). Thermal stabilization effect of (*a*) chemically modified heparins (table 1, D2–D9), and other glycosaminoglycan (HS, HA, CS-C and DS), (*b*) heparin-derived oligosaccharides, ranging from dp2 to dp12 and (*c*) cation-modified heparin forms. Results are the mean of triplicates after normalization ± s.e.

FGF3, a member of the FGF7 subfamily according to amino acid sequence alignment, was similarly stabilized by unmodified heparin and any of the singly desulfated heparins (table 1, D2–D4; figure 2*a*). However, the doubly desulfated heparins with just a 6-*O*-sulfate or *N*-sulfate (table 1, D5 and D7) stabilized FGF3 to 40% and 25% of the level observed with heparin, respectively, whereas heparin with just 2-*O*-sulfate was without a detectable effect. Totally desulfated heparin was also without effect. FGF3 was most stabilized by persulfated heparin, whereas HS was as effective as the doubly desulfated *N*-sulfated or 6-*O* sulfated heparins, D5 and D6. FGF3 did not have a detectable interaction with HA or CS-C, but DS clearly did bind, albeit not as well as HS. A dp4 was the shortest oligosaccharide able to stabilize FGF3 with a maximum effect at dp10, which stabilized FGF3 to an extent similar to full-length heparin (figure 2*b*). FGF3 did not discriminate between the different cationic forms of heparin, because these all had the same stabilizing effect (figure 2*c*). Thus, FGF3 has a clear preference for a saccharide structure with any two of *N*-, 2-*O* and 6-*O* sulfate and is able to bind structures with doubly desulfated heparin containing just a 6-*O*-sulfate or an *N*-sulfate, whereas a dp10 is likely to represent the full-length binding structure in the polysaccharide.

**Figure 3.**
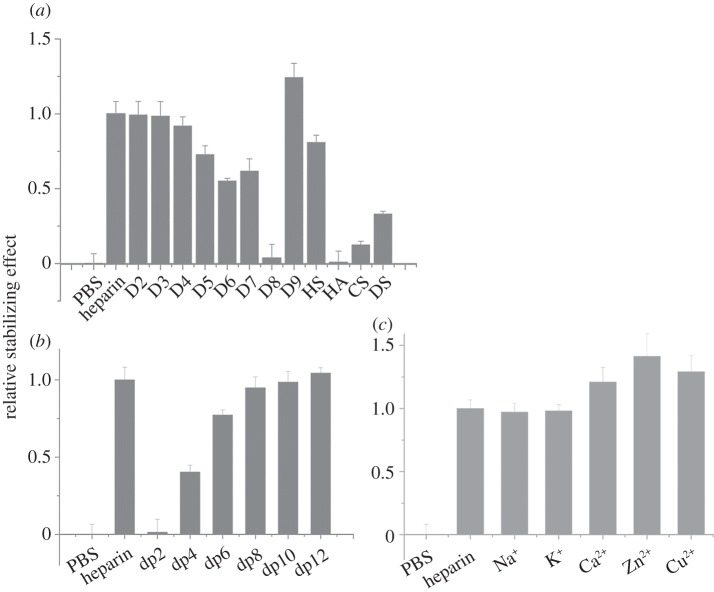
Differential scanning fluorimetry (DSF) analysis of binding of glycosaminoglycan derivatives to FGF10. DSF of 5 µM FGF10 was performed in the presence of a range of heparin-based poly- and oligosaccharides (all 10 µM) and the thermal stabilization relative to the PBS control (=0) and heparin (=1) was calculated (see ‘Differential scanning fluorimetry’). Thermal stabilization effect of (*a*) chemically modified heparins (table 1, D2–D9), and other glycosaminoglycan (HS, HA, CS and DS), (*b*) heparin-derived oligosaccharides, ranging from dp2 to dp12 and (*c*) cation-modified heparin forms. Results are the mean of triplicates after normalization ± s.e.

In the case of FGF10, another member of the FGF7 subfamily, there was no discernible difference in the stabilizing effect of heparin and the singly desulfated heparins (table 1, D2–D4; figure 3*a*). However, all three doubly desulfated heparins (D5–D7) had a similar stabilizing effect, which was approximately 50–70% of that seen with heparin. Totally desulfated heparin and HA failed to bind FGF10, whereas FGF10 bound persulfated heparin more effectively than heparin and HS only slightly more weakly. FGF10 also bound both CS-C and DS, though the former more weakly. A heparin-derived dp4 oligosaccharide provided substantial binding and maximum binding was seen with a dp8, indicating that this is the likely minimum-sized fragment of the polysaccharide required for interaction. FGF10 may also have a slight preference for the Ca^2+^, Zn^2+^ and Cu^2+^ cation forms of heparin. Thus, the binding preferences of FGF10 are similar but not identical to those of FGF3. Compared with FGF3, FGF10 has a less marked preference for singly over doubly desulfated heparins, it does not appreciably distinguish between any of the three doubly desulfated heparins, and has a wider range of glycosaminoglycan species (CS-C as well as DS) with which it can interact (figures 1*b*,*c* and 2*b,c*).

**Figure 4.**
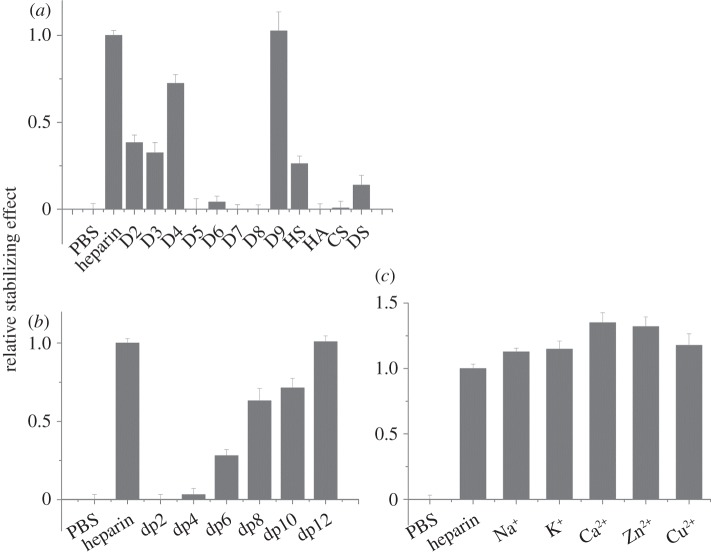
Differential scanning fluorimetry (DSF) analysis of binding of glycosaminoglycan derivatives to FGF4. DSF of 5 µM FGF4 was performed in the presence of a range of heparin-based poly- and oligosaccharides (all 10 µM) and the thermal stabilization relative to the PBS control (=0) and heparin (=1) was calculated (see ‘Differential scanning fluorimetry’). Thermal stabilization effect of (*a*) chemically modified heparins (table 1, D2–D9), and other glycosaminoglycan (HS, HA, CS, and DS), (*b*) heparin-derived oligosaccharides, ranging from dp2 to dp12 and (*c*) cation-modified heparin forms. Results are the mean of triplicates after normalization ± s.e.

For FGF4, there was a greater thermal stabilization by heparin than by any of the singly desulfated heparins (figure 4*a*). Moreover, among the singly desulfated heparins, FGF4 had a preference for polysaccharides with both 2-*O* and *N*- sulfate (D4) over polysaccharides with *N*-sulfate and 6-*O* sulfate (D3), or 2-*O* and 6-*O* sulfate (D2). There was no appreciable effect of the doubly desulfated heparins on the thermal stability of FGF4. These data suggest that the core recognition structure of FGF4 in the polysaccharide involves a 2-*O* and *N*-sulfated structure. The lower stabilization observed with HS may reflect that sequences of the appropriate length containing this motif are relatively rare in this material. FGF4 also bound DS, though weakly, but did not bind to HA or CS-C (figure 4*a*). FGF4 did not interact detectably with a dp4, and a dp6 was the minimal fragment required for binding. Maximal binding, equivalent to that observed with heparin, was achieved with a dp12 (figure 4*b*). Little effect was observed for the different cation coordinated forms of heparin, indicating that this parameter, which changes the conformation of the polysaccharide chain [40], does not, at least in the case of the heparin polysaccharide, influence the binding of FGF4 (figure 4*c*).

**Figure 5.**
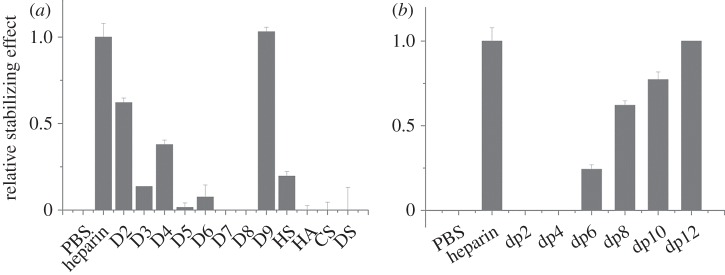
Differential scanning fluorimetry (DSF) analysis of binding of glycosaminoglycan derivatives to HT-FGF6. DSF of 5 µM HT-FGF6 was performed in the presence of a range of heparin-based poly- and oligosaccharides (all 10 µM) and the thermal stabilization relative to the PBS control (=0) and heparin (=1) was calculated (see ‘Differential scanning fluorimetry’). Thermal stabilization effect of (*a*) chemically modified heparins (table 1, D2–D9), and other glycosaminoglycan (HS, HA, CS, and DS) and (*b*) heparin-derived oligosaccharides, ranging from dp2 to dp12. Results are the mean of triplicates after normalization ± s.e., and an apparent absence of error bar is due to a small s.e.

FGF6 is another member of the FGF4 subfamily, and HT-FGF6 was more effectively stabilized by heparin than the singly desulfated heparins (figure 5*a*). Of the latter, heparins with a 2-*O*-sulfated iduronate bound better than D3, which lacks this sulfate group, and there was a slight preference for D2 (GlcNAc, 6S, IdoA 2S) over D4 (GlcNS, IdoA 2S; figure 5*a*). With the exception of 2-*O*-sulfated heparin, the doubly desulfated heparins did not have a measureable stabilization effect on HT-FGF6, which highlights the preference of FGF6 for a structure containing 2-*O*-sulfate (figure 5*a*). HT-FGF6 bound persulfated heparin as effectively as native heparin, but its interaction with HS was similar to that with the singly desulfated heparin lacking Ido2S. No binding to HA, CS-C or DS was detected (figure 5*a*). The minimum size and maximum size of oligosaccharide required for binding to FGF6 was dp6 and dp12, respectively (figure 5*b*).

**Figure 6.**
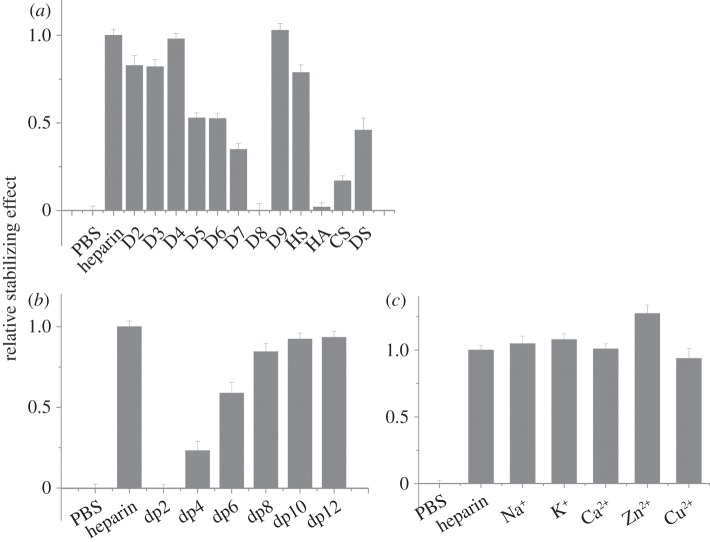
Differential scanning fluorimetry (DSF) analysis of binding of glycosaminoglycan derivatives to FGF17. DSF of 5 µM FGF17 was performed in the presence of a range of heparin-based poly- and oligosaccharides (all 10 µM) and the thermal stabilization relative to the PBS control (=0) and heparin (=1) was calculated (see ‘Differential scanning fluorimetry’). Thermal stabilization effect of (*a*) chemically modified heparins (table 1, D2–D9), and other glycosaminoglycan (HS, HA, CS, and DS), (*b*) heparin-derived oligosaccharides, ranging from dp2 to dp12 and (*c*) cation-modified heparin forms. Results are the mean of triplicates after normalization ± s.e.

FGF17 bound the three singly desulfated heparins similarly to heparin and the doubly desulfated heparins more weakly (figure 6*a*). Moreover, FGF17 had a mild preference for heparin with either a 2-*O* or a 6-*O* sulfate, compared with heparin with just an *N*-sulfate (figure 6*a*). FGF17 did not bind desulfated heparin or HA, but bound persulfated heparin and HS similarly to heparin. It also interacted with DS to a similar extent as the singly desulfated heparins and more weakly with CS-C. FGF17 required at least a dp4 oligosaccharide for binding and maximal binding was observed with a dp8. It might have a slight preference for Zn^2+^ coordinated heparin over other cationic forms of the polysaccharides (figure 6*b,c*).

**Figure 7.**
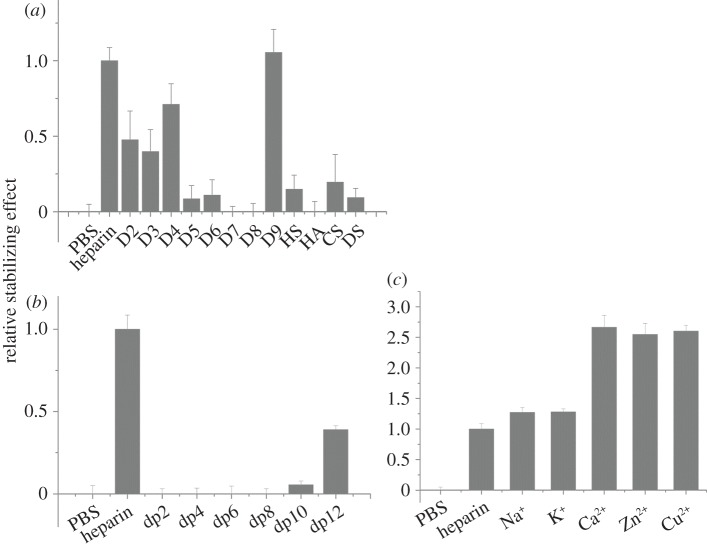
Differential scanning fluorimetry (DSF) analysis of binding of glycosaminoglycan derivatives to FGF20. DSF of 5 µM FGF20 was performed in the presence of a range of heparin-based poly- and oligosaccharides (all 10 µM) and the thermal stabilization relative to the PBS control (=0) and heparin (=1) was calculated (see ‘Differential scanning fluorimetry’). Thermal stabilization effect of (*a*) chemically modified heparins (table 1, D2–D9), and other glycosaminoglycan (HS, HA, CS and DS), (*b*) heparin-derived oligosaccharides, ranging from dp2 to dp12 and (*c*) cation-modified heparin forms. Results are the mean of triplicates after normalization ± s.e.

Heparin was more effective at stabilizing FGF20 than any of the singly desulfated heparins (figure 7*a*). FGF20 had a weak interaction with the doubly desulfated heparin possessing just 6-*O*-sulfate or 2-*O*-sulfate, whereas there was no detectable interaction with heparin possessing just an *N*-sulfate, which suggests a preference for the former two sulfation positions. The stabilizing effects of CS-C and DS on FGF20 were similar to that seen with HS. The minimum size of oligosaccharide required for binding to FGF20 was dp10, whereas the maximum size of oligosaccharide used in this assay, dp12, only stabilized the protein to around 40% of the extent observed with heparin (figure 7*b*). The binding of FGF20 to the polysaccharide was markedly affected by the coordinating cations: the divalent cation (Ca^2+^, Zn^2+^ or Cu^2+^) coordinated heparins were twice as effective in stabilizing FGF20 as heparins coordinated to a monovalent cation (Na^1+^ and K^1+^; figure 7*c*).

### 3.3. Identification of lysines involved in heparin binding by protect and label

The lysine residues involved in binding heparin in the FGFs were determined by the ‘protect and label’ approach, where lysines in binding sites are protected with acetyl groups, when the FGFs are bound to heparin [34]. Following release of the FGF from heparin, the newly exposed lysines that had been involved in binding were labelled with biotin and identified by mass spectrometry. The nomenclature of the HBSs is that used previously [31,34], where the canonical heparin binding site is HBS1, and the secondary sites are HBS2–4.

### 3.4. FGF7 subfamily (FGF3/FGF10)

**Figure 8.**
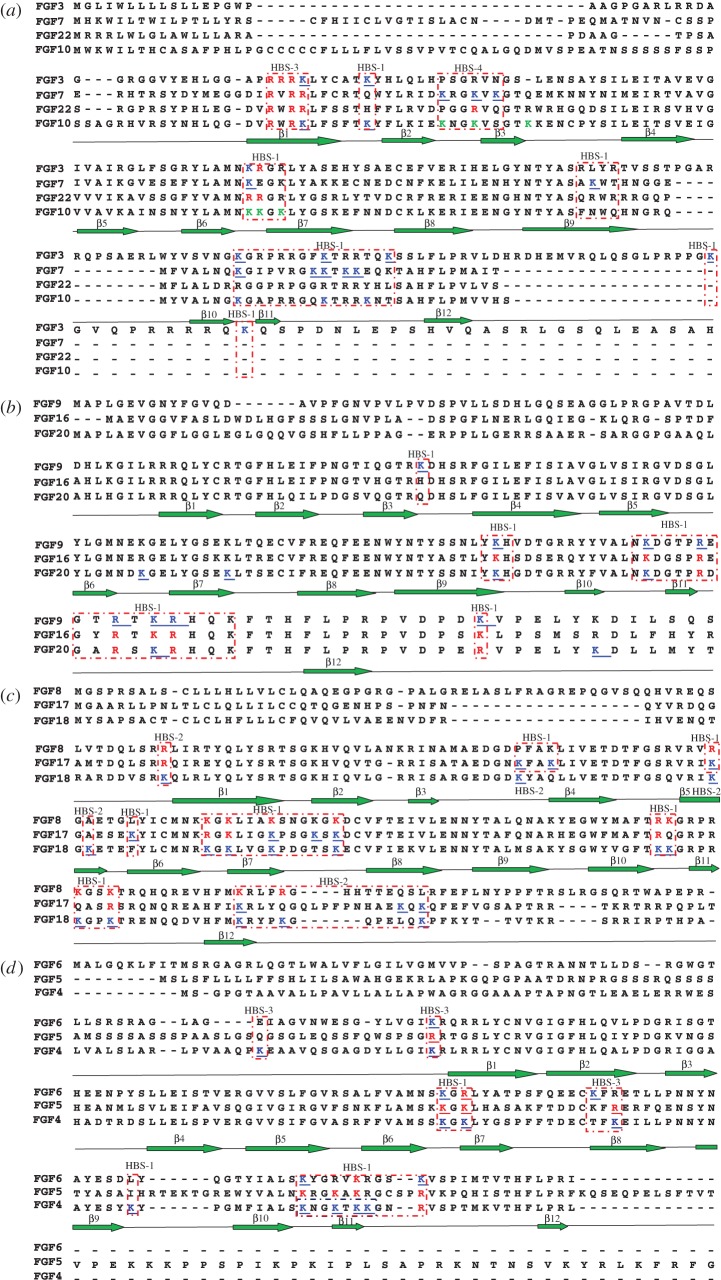
Sequence alignment of human FGF subfamilies. The sequences were aligned with ClustalX and Dendroscope [41,42].

Initial experiments with FGF3 and FGF10 identified just one peptide with biotinylated lysines, Lys-47 in FGF3 and Lys-81 in FGF10 both in strand β1 (table 2). This was considered to be due to the use of chymotrypsin to cleave the protein, which may produce peptides from these FGFs that are either too long or too short for detection with MALDI-MS. To identify further peptides, FGF3 and FGF10 were digested with trypsin (cleaves at Arg residues only, owing to the protect and label procedure) and thermolysin (cleaves at Pro, His, Asp, Glu residues), as modifications of the published method [34]. By changing the protease used to cleave the lysine-modified protein, a substantial number of biotinylated lysine residues were identified in FGF3. Lys-160, Lys-168, Lys-174, Lys-204 and Lys-214 were found to be biotinylated. These residues are located in the loops between strands β10 and β11 and between strands β11 and β12 and C-terminal to strand β12. They correspond to the HBS1 of FGF3, predicted by sequence alignment (figure 8) [31]. In addition, two other lysine residues, which are close to the above residues of the canonical HBS1, were biotin-labelled: Lys-53, which lies between strands β1 and β2, and Lys-101, which is between strands β6 and β7. Thus, these two residues are likely to be part of the canonical binding site, which, therefore, has contributions from residues that are distant in the primary sequence, but neighbouring in the folded protein. However, biotinylated Lys-47 on strand β1 is distant from the canonical binding site. Along with the neighbouring arginine (Arg-44–46), this would be part of the secondary binding site termed HBS3 in FGF2 [34]. The amino acids in FGF3 corresponding to HBS4 identified in FGF7 are arginine and asparagine (figure 8; [31]), which would not be detected by the lysine-targeted protect and label used here. Thus, FGF3 may also possess an HBS4, but this remains to be established.

**Table 2.** Summary of peptides of FGF3 and of FGF10 identified by lysine-targeted protect and label. Labelled peptides were identified by MALDI-Q-TOF and analysed by MS-digest from the package ProteinProspector v. 5.12.3. A full list of identified peptides is provided in the electronic supplementary material, table S1. The three proteases used for protein digestion were trypsin (TRY), thermolysin (THE) and chymotrypsin (CHY). The spectrums were shown in the electronic supplementary material, figure S7–S11.

	peptide	sequence	protease	residue	HBS	spectrum
FGF3	1	ATK(biotin)YHLQ	(THE)	51–57	1	S8
	2	AMNK(biotin)RGR	(THE)	98–104	1	S8
	3	LWYVSVNGK(biotin)GRPR	(TRY)	152–164	1	S7
	4	RGFK(biotin)TR	(TRY)	165–170	1	S7
	5	TQK(biotin)SSLFLPR	(TRY)	172–181	1	S7
	6	QLQSGLPRPPGK(biotin)GVQPR	(TRY)	193–209	1	S7
	7	QK(biotin)QSPDNLEPSHVQASR	(TRY)	213–229	1	S7
	8	EHLGGAPRRRK(biotin)L	(CHY)	37–48	4	S9
FGF10	1	QMYVALNGK(biotin)GAPR	(TRY)	175–187	1	S10
	2	RGQK(biotin)TR	(TRY)	188–193	1	S10
	3	K(biotin)NTSAHFLPMVVHS	(TRY)	195–208	1	S10
	4	RK(biotin)LFSFTK(biotin)Y	(CHY)	80–88	1/3	S11
	5	IEKNGKVSGTK (2xbiotin)	(TRY)	92–102	4	S10
	6	YLAMNK(biotin/acetyl)K(biotin/acetyl)GK(biotin/acetyl)LY	(CHY)	131–141	4	S11

**Figure 9.**
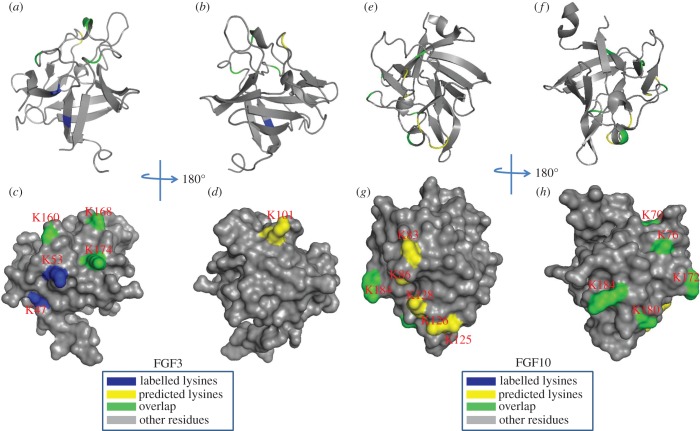
Position of biotinylated lysines in FGF3 (residues 56–202) and in FGF10 (69–207) identified by structural proteomics mapped onto their predicted three-dimensional structures. The published structure of FGF10 (69–207; PDB 1NUN) [43] was used to generate a model of the structure of FGF3. Labelled lysines are coloured in blue and literature annotated and/or predicted lysines in HBS1 are coloured in yellow. The lysines overlapping with the literature annotated and predicted aligned canonical HBS1 lysines are coloured in green. (*a,b,e,f*) Ribbon diagram; (*c,d,g,h*) corresponding molecular surface. (*b*,*d*) 180° back view of (*a,c*); (*f,h*), 180° back view of (*e,g*).

Lys-87, Lys-184, Lys-191 and Lys-195 were all labelled in FGF10 (table 2). These lysine residues are in the canonical HBS1 of FGF10, as predicted by sequence alignment (figure 9). Three lysines of the peptide ‘^131^YLAMNKKGKLY^141^’ (Lys-125, Lys-126 and Lys-128) of FGF10 were found to be both acetylated and biotinylated (figure 9). This has been observed previously in other proteins [34,44], and is considered to be due to the local dissociation of a lysine side chain from its interaction with the polysaccharide. In the presence of the NHS–acetate used in the protection step, the transiently dissociated lysine side chain becomes acetylated, and this would likely preclude its re-binding to the polysaccharide. Because the protein remains bound, these lysines must form part of the binding site that is relatively dynamic over the timescale of the protection step, but the remainder of the binding site is not dynamic, such that the FGF10 remains bound to the heparin column. Two biotinylated lysines were also identified in peptide ‘IEKNGKVSGTK’ (residues 92–102). Owing to the position of these residues on the surface of FGF10, they are most likely to form part of HBS4, as identified in FGF7 previously and inferred in FGF3 [31] (figure 9). HBS4 lies orthogonal to HBS1 in all three proteins, thus the binding of the polysaccharide to these two sites is likely to be mutually exclusive. Similar to FGF3, the biotinylated Lys-81 located in strand β1 of FGF10 has also been identified to be part of HBS3. Because the aligned HBS3 on FGF7 has arginine residues rather than lysines, it was not detected [31]. However, the identification of HBS3 in FGF3 and FGF10 strongly suggests that the corresponding sequence in FGF7 has the same function.

### 3.5. FGF4 subfamily (FGF4/FGF6)

**Figure 10.**
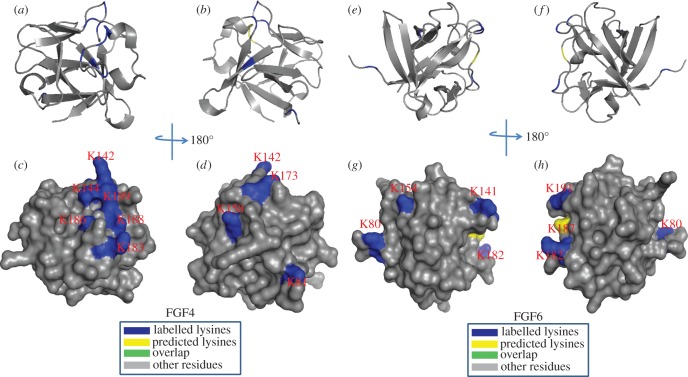
Position of biotinylated lysines in FGF4 (residues 79–206) and in FGF6 (residues 47–174) identified by structural proteomics mapped onto their predicted three-dimensional structures. The published structure of FGF4 (79–206; PDB 1IJT) [45] was used to generate a structure of FGF6. Labelled lysines are coloured in blue and literature annotated and/or predicted lysines in HBS1 are coloured in yellow. The lysines overlapping with the literature annotated and predicted aligned canonical HBS1 lysines are coloured in green. (*a,b,e,f*) Ribbon diagram; (*c,d,g,h*) corresponding molecular surface. (*b,d*) 180° back view of (*a,c*); (*f,h*), 180° back view of (*e,g*).

For FGF4, Lys-183, Lys-186, Lys-188 and Lys-189 in the area between the β10 strand and β12 strand were found to be biotinylated (table 3). These lysine residues correspond to the HBS1 of FGF4 predicted by sequence alignment (figure 10). The mutation to alanine of Lys-183 and Lys-188 in FGF4 had previously identified these residues as being part of the canonical HBS1 [45]. Moreover, another three labelled lysine residues are physically adjacent: Lys-142 is in the loop between the β6 strand and β7 strand; Lys-144 is on the β7 strand; Lys-147 is in the loop between the β9 strand and β10 strand. These six residues can be considered to delineate the canonical HBS1 of FGF4 (figure 10). Further biotinylated lysines (Lys-65, Lys-81 and Lys-158) were identified. They are aligned with a secondary HBS, HBS3, in other FGFs (figure 8) [31,34]. Similar to FGF4, FGF6 has two HBSs: HBS1, which is identified by biotinylated Lys-144, Lys-185 and Lys-194 (separately, loop between β6 strand and β7 strand, area between β10 strand and β12 strand); and HBS3, which includes Lys-83 towards the N-terminal of strand β1 and Lys-158 on the β8 strand (figure 10; tables 4 and 5).

**Table 3.** Summary of peptides of FGF4 and of FGF6 identified by lysine-targeted protect and label. Labelled peptides were identified by MALDI-Q-TOF and analysed by MS-digest from the package ProteinProspector v/ 5.12.3. A full list of identified peptides is provided in the electronic supplementary material, table S1. The three proteases used for protein digestion were trypsin (TRY), thermolysin (THE) and chymotrypsin (CHY). The spectrums were shown in the electronic supplementary material, figure S12–S16.

	peptide	sequence	protease	residue	HBS	spectrum
FGF4	1	VAMSSK(biotin)GK(biotin)LY	(CHY)	137–146	1	S12
	2	LPNNYNAYESYK(biotin)YPGMF	(CHY)	162–178	1	S12
	3	LSK(biotin)NGK(biotin)TK(biotin)K(biotin)GNRVSPT	(THE)	181–196	1	S13
	4	AQPK(biotin)EAAVQSGAGDY	(THE)	62–76	3	S14
	5	LLGIK(biotin)RL	(CHY)	77–83	3	S12
	6	FK(biotin)EILLPNNYN	(THE)	157–167	3	S14
FGF6	1	SALFVAMNSK(biotin)GR	(TRY)	135–146	1	S15
	2	IALSK(biotin)Y	(CHY)	181–186	1	S16
	3	GSK(biotin)VSPIMTVTHFLPR	(TRY)	192–207	1	S15
	4	SRAGLAGEIAGVNWESGYLVGIK(biotin)RQRR	(TRY)	61–87	3	S15
	5	LYATPSFQEEC(carbamidomethyl)K(biotin)FR	(TRY)	147–160	3	S15

**Table 4.** Summary of peptides of FGF17 identified by lysine-targeted protect and label. Labelled peptides were identified by MALDI-Q-TOF and analysed by MS-digest from the package ProteinProspector v. 5.12.3. A full list of identified peptides is provided in the electronic supplementary material, table S1. The three proteases used for protein digestion were thermolysin (THE), chymotrypsin (CHY) and Glu-C (GLU). The spectrums were shown in the electronic supplementary material, figure S17–S21.

	peptide	sequence	protease	residue	HBS	spectrum
FGF17	1	GNK(biotin)FAK(biotin)LIVETD	(GLU)	80–91	1	S17
	2	GSRVRIK(biotin)GAESEK(biotin)Y	(CHY)	94–107	1	S18
	3	LIGK(biotin)PSGK(biotin)SK(biotin)DCVFTE	(THE)	116–131	1	S19
	4	IK(biotin)RLY	(CHY)	175–179	2	S18
	5	QGQLPFPNHAEK(biotin)QK(biotin)QF	(CHY)	180–195	2	S18

**Table 5.** Summary of peptides of FGF20 identified by lysine-targeted protect and label. Labelled peptides were identified by MALDI-Q-TOF and analysed by MS-digest from the package ProteinProspector v. 5.12.3. A full list of identified peptides is provided in the electronic supplementary material, table S1. The two proteases used for protein digestion were thermolysin (THE) and chymotrypsin (CHY). The spectrums were shown in the electronic supplementary material, figure S22–S23.

	peptide	sequence	protease	residue	HBS	spectrum
FGF20	1	YLGMNDK(biotin)GEL	(CHY)	118–127	1	S22
	2	YGSEK(biotin)LTSECIF	(CHY)	128–139	1	S22
	3	K(biotin)HGDTGRRYF	(CHY)	157–166	1	S22
	4	LNK(biotin)DGTPRDGARSK(biotin)RHQK(biotin)FTH	(THE)	169–189	1	S23
	5	K(biotin)DLLMYT	(CHY)	205–211	3	S22

### 3.6. FGF8 subfamily (FGF17)

**Figure 11.**
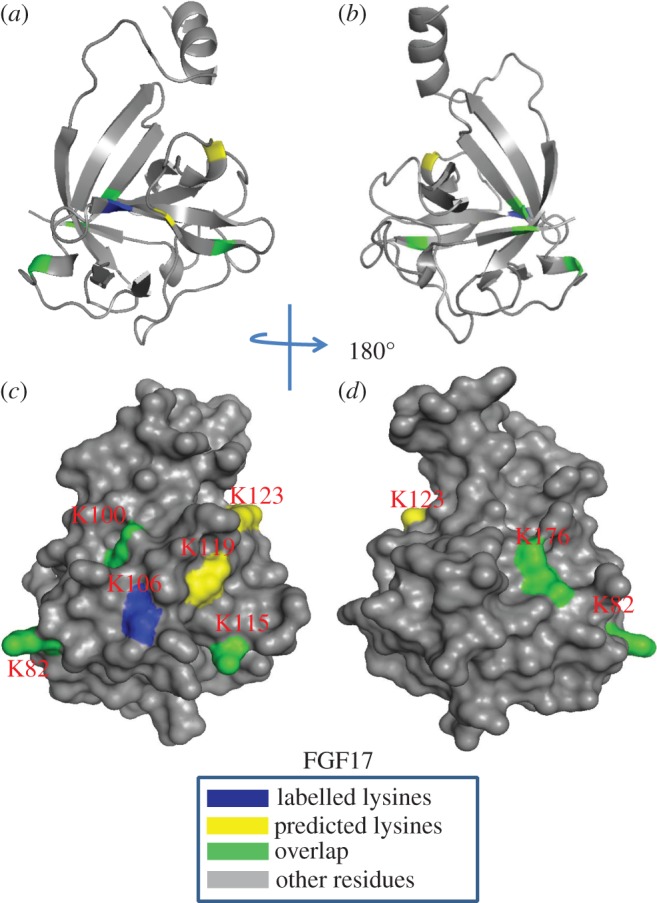
Position of biotinylated peptides in FGF17 (residues 33–178) identified by structural proteomics mapped onto their predicted three-dimensional structure. The published structure of FGF8 (PDB 2FDB) [46] was used to generate a structure of FGF17. Labelled lysines are coloured in blue and literature annotated and/or predicted lysines in HBS1 are coloured in yellow. The lysines overlapping with the literature annotated and predicted aligned canonical HBS1 lysines are coloured in green. (*a,b*) Ribbon diagram; (*c,d*) corresponding molecular surface. (*b,d*) 180° back view of (*a,c*).

In the case of FGF17, the predicted canonical HBS1 contains arginine but no lysine residues and, therefore, no peptides were identified between strands β10 and β12. However, Lys-82 and Lys-85, located in the loop between strands β3 and β4, Lys-100 on strand β5, Lys-106 on strand β6 and Lys-119, Lys-123 and Lys-125 on strand β7 and the loop between strands β7 and β8 were all found to be biotinylated. These residues are physically adjacent to the region between strand β10 and β12, where the core of the HBS1 of FGFs is predicted to be located. In addition, FGF17 has an HBS2, which includes Lys-176, Lys-191 and Lys-193 at the C-terminus. The aspartic acid (Asp-121) of FGF18 enlarges the negative border formed by two glutamic acid residues (Glu-103 and Glu-105), the result of which is that Lys-82 and Lys-100 are part of the extended HBS2. In contrast, Asp-121 of FGF18 is Ser-121 in FGF17, which consequently has a smaller negatively charged border along its HBS1 and Lys-82 and Lys-100 are now likely to be part of an extended HBS1. These data demonstrate how changes in the residues surrounding HBS1 (in this instance Asp to Ser) can alter the structure of an HBS and provide subtle difference between members of the same subfamily (figure 11).

### 3.7. FGF9 subfamily (FGF20)

**Figure 12.**
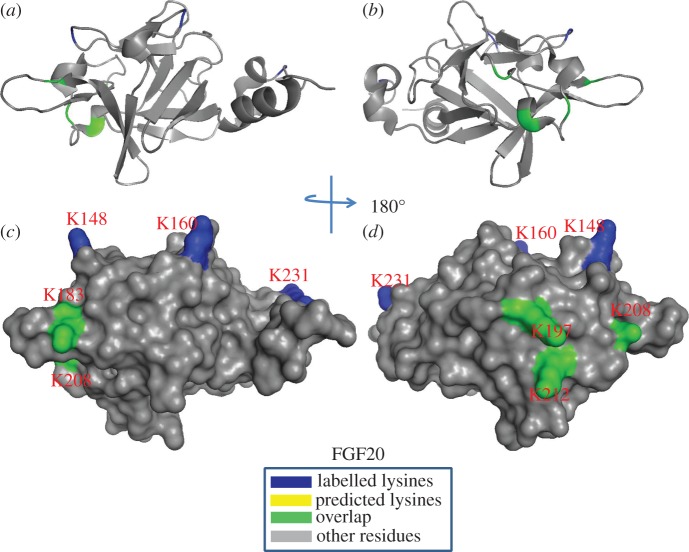
Position of biotinylated peptides in FGF20 (residues 33–178) identified by structural proteomics mapped onto three-dimensional structure. The published structure of FGF20 (residues 33–178; PDB 3F1R) was used [47]. Labelled lysines are coloured in blue and literature annotated and/or predicted lysines in HBS1 are coloured in yellow. The lysines overlapping with the literature annotated and predicted aligned canonical HBS1 lysines are coloured in green. (*a,b*) Ribbon diagram; (*c,d*) corresponding molecular surface. (*b,d*) 180° back view of (*a,c*). Labelled lysines and those overlapped with aligned HBS lysines are coloured in blue, predicted lysines and arginines are coloured in red. Lysines that were both acetylated and biotinylated are coloured in green. β-strands are according to one structure in each subfamily (FGF10, PDB 1NUN; FGF20, PDB 3F1R; FGF8, 2FDB; FGF4, PDB 1IJT).

For FGF20, Lys-183, Lys-197, Lys-208 and Lys-212 located in strand β9, the loop between strand β10 and strand β11, and the loop between strand β11 and strand β12 were biotinylated and these correspond to the predicted HBS1 by sequence alignment [31]. Two further lysine residues (Lys-148 and Lys-183) that are located in the area between strand β6 and strand β7 were also identified to be biotinylated. Because these two lysines are physically adjacent to the canonical binding site, they were considered to be an extension of the HBS1. The biotinylated Lys-231 located in the C-terminus is quite close to Arg-90, Arg-91 and Arg-92, which may form the HBS3 (figure 12), although, arginine residues cannot be identified by the NHS chemistry used here. In any event, FGF20 possesses a single, enlarged HBS-1, similar to FGF9 [31], but unlike FGF9 it may also possess an HBS3. The equivalent residue in FGF9, Lys-202, was not found to be labelled in previous work [31], which may be due either to only one protease being used in this work or to the existence of a very well defined negatively charged border around this lysine and the three physically adjacent arginine residues in FGF20. FGF20 has been found to exist as a non-covalent dimer in solution [39,47], which is also true for the protein we have produced (electronic supplementary material, figure S6). When the enlarged HBS1 is mapped onto the dimer structure, the HBS1 from both FGF20 monomers are joined to form a single large heparin binding surface.

## 4. Discussion

To resolve the extent to which molecular specificity underpins the interactions of proteins with HS, we have used the phylogenetic relationship of the FGF family, established from amino acid sequence, as a model system. The interactions of FGFs and heparin/HS have been measured from two perspectives: the structures in the polysaccharide required for binding and the lysines in HBSs on the FGFs.

**Figure 13.**
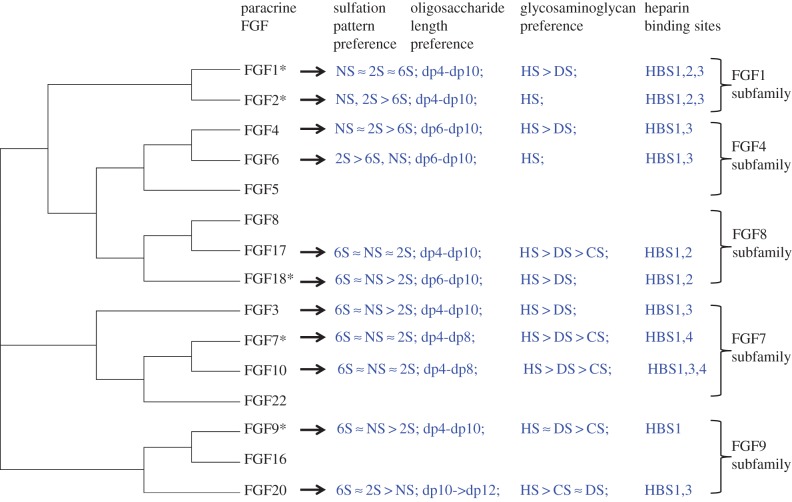
The heparin structural preference of FGFs and their heparin binding sites. Asterisk denotes data from [31,33,34]. The preference of FGF members from different subfamilies for the sulfation pattern, oligosaccharide length, glycosaminoglycan and binding sites of heparin structure.

Taking the present results with previous ones [31,33,34] provides the first comprehensive coverage of the structural basis of the interactions of a protein family with sulfated polysaccharide structures. These data collectively encompass 11 paracrine FGFs, all of which bind HS, with at least two FGFs from each of the subfamilies. The data follow a clear pattern, which is not related to the overall charge density of the polysaccharide (table 1 and Material and methods). FGF members from the same subfamily have preference for binding polysaccharide structures with similar patterns of sulfation and length, whereas FGFs from different subfamilies have much more pronounced differences in these preferences (figure 13). Moreover, FGFs from the same subfamily possess similar secondary HS binding sites and their primary HBS1 have similar architectures. Again, FGFs from different subfamilies have different combinations of secondary HBS and their HBS1 differs, particularly with respect to the extent to which amino acids that are distant in sequence, but physically close, contribute to the HBS1 (figure 13). Thus, in the FGF1 subfamily, both FGF1 and FGF2 have similar preference for *N*-sulfate and 2-*O*-sulfate, but FGF1 differs in that it also binds saccharide structures with 6-*O*-sulfated heparin [33]. This subfamily possesses three HBSs, the primary HBS1 and the secondary binding sites HBS2 and HBS3 [31,34,48]. In the FGF4 subfamily, FGF4 prefers structures containing 2-*O* and *N*-sulfate, whereas FGF6 binds strongly to structures with 2-*O* and either 6-*O*- or NS. Compared with FGF4, FGF6 needs a slightly larger structure for minimum binding (dp6). Both FGF4 and FGF6 have a single secondary binding site, which would correspond to HBS3 in the FGF1 subfamily. In the case of the FGF7 subfamily, FGF7 and FGF10 have preference for a similar pattern of sulfation and oligosaccharide length. However, FGF3 barely binds to doubly desulfated heparin containing only 2-*O*-sulfate and it required large structures for full binding. The putative HBS3 of FGF7 and HBS4 of FGF3, identified by amino acid sequence alignment, contain arginine but not lysine [31] (figure 8), so cannot be identified by our lysine-targeted method. The protect and label data, when combined with sequence alignment (figure 8), indicate that the FGF7 family possesses two secondary HBSs, HBS3 and HBS4, the latter being physically orthogonal to the canonical HBS1. FGF17 and FGF18, which are in the FGF8 subfamily, bind to similar structures containing 6-*O*-sulfate and *N*-sulfate and they contain a single secondary binding site, HBS2. In the FGF9 subfamily, FGF9 and FGF20 show a similar preference for 6-*O*-sulfated heparin. Whereas FGF9 prefers to bind to structures containing *N*-sulfate rather than 2-*O*-sulfate, FGF20 binds strongly to 2-*O*-sulfated heparin. Although FGF20 required larger structures for binding than FGF9, this could be caused by its dimeric structure [39,47]. Both FGF9 and FGF20 possess a single, enlarged HBS1. A secondary HBS3 was also been found in FGF20; whereas corresponding basic residues are present in FGF9, in the latter, there is no negatively charged border and so they are more likely to be part of an extended HBS1. In contrast, in FGF20, these residues are surrounded by a negative border, which would isolate them. Consequently, FGF20 seems likely to have a distinct HBS3 and may, therefore, unlike FGF9, be able to cross-link HS chains [49].

The expansion of the FGF family and its divergence into subfamilies occurred through genome duplication events that led to even more complex animal body plans and physiology [19]. The present analysis indicates that the molecular specificity of the FGFs for particular structures in HS and the pattern of secondary binding sites on the FGFs also underwent a similar diversification. This implies that the molecular basis of the interaction of FGFs with glycosaminoglycan has been subjected to the same natural selection processes that gave rise to an expanded FGF family, which is similar to what is seen with respect to the specificity of FGFs for their receptor tyrosine kinases (FGFR) [50,51] and borne out by an analysis of the interactions of nine paracrine FGFs [32]. Therefore, the differences we observe in the structural basis of FGF–glycosaminoglycan interactions (figure 13) are likely to be linked to the functional differences that exist between FGFs and between their subfamilies. This has important ramifications in relation to our understanding of protein–glycosaminoglycan interactions. It is interesting to note that *Caenorhabditis elegans* and *Drosophila* possess far fewer FGFs than mammals (two and three, respectively) and synthesize simpler HS structures [52], though whether there is a general link between the expansion of HS binding proteins and more complex glycosaminoglycan biosynthesis in evolution remains to be established.

The molecular specificity (figure 13) is far from absolute. For example, there is a consensus ranking of sulfations, oligosaccharide length and glycosaminoglycan preference for FGFs in the same subfamily, but this is not a simple one-to-one code (figure 13). This raises the question of how specific and selective protein–glycosaminoglycan interactions are, to which there have been varied answers [30,53]. In this respect, excellent binding structures in sulfated polysaccharides that are unrelated to glycosaminoglycan have been identified for some FGFs [54]. This supports the contention that it is the spatial disposition of sulfate, carboxyl and hydroxyl groups on the polysaccharide that are important for binding. The sugar chain will adopt a variety of conformations in solution and pendant sulfate groups will modify the conformational space that the chain can occupy, which has been demonstrated by NMR and CD studies [55]. In addition, the coordination of cations modifies the conformation of the polysaccharide chain [36]. Finally, while the binding to polysaccharide clearly changes the conformation of the protein, e.g. thermal stabilization observed by DSF, the reverse is also true: binding to protein alters the conformation of the polysaccharide. The latter point is elegantly made by the co-crystal structure of FGF2 and a heparin dp6, in which the latter has iduronate residues in both the ^1^C_4_ and the ^2^S_0_ configurations [56]. Thus, the selectivity and specificity identified here is somewhat artificial, because the conformation of HS *in vivo* in extracellular and pericellular matrix will depend on the sequence of saccharides, the coordinated cations and the pre-existing interactions of the HS chain with endogenous proteins.

It is intriguing that at some level cells can sense what functional structures they produce and modify these. This is shown by the HS 2-*O* sulfotransferase knockout mouse, which dies at birth owing to kidney agenesis [57]. HS or heparin lacking 2-*O*-sulfate cannot bind FGF2 or form a productive ternary complex with the FGFR [58]. Yet, the knockout mice have no FGF2 phenotype [57]. Moreover, when embryonic fibroblasts were derived from these mice, their HS did not possess any 2-*O*-sulfated HS, but the HS was capable of interacting with FGF2 and enabling it to bind and activate FGFR on cells [59]. Thus, there would appear to be homeostatic mechanisms whereby cells can modify the chains they produce and perhaps cations coordinated to HS and/or the endogenous proteins bound to this HS, to ensure that as many as possible of the appropriate functions are maintained after perturbation. Such homeostatic plasticity can be considered to be advantageous, because it provides for a robust rather than a brittle regulation of cell communication. However, this clearly limits the degree to which one can apply simple interpretations to the molecular basis of specificity of FGF–glycosaminoglycan interactions. This is likely to be true of protein–glycosaminoglycan interactions in general. For example, in addition to its classic pentasaccharide binding sequence [5], which has been the underpinning of arguments for absolute specificity of protein–HS interactions, there are good antithrombin III binding structures with anticoagulant activity that are substantially different [27,28].

The evolutionary divergence and, within FGF subfamilies, conservation of HS binding properties, indicates that these have functional importance. This is demonstrated by the requirement for HS as a co-receptor for the formation of the classic growth-stimulatory signalling complex with the FGFR [6,7]. However, HS binding has a range of other functions, one of which is the regulation of the diffusion of FGFs in the extracellular matrix. Thus, the measurement of the diffusion of FGFs in the pericellular matrix of fibroblasts [8,60] shows that the diffusion properties of FGFs are determined at least in part by their binding specificities for glycosaminoglycans. It is established that HS controls the transport and diffusion of other HS-binding effectors, for example, in development where HS binding influences morphogen gradients [13,61,62] and in guiding immune cells [63]. The selectivity of HS for different proteins, demonstrated here across the FGF family, may enable differential yet simultaneous control of the bioavailability and of gradients of numerous HS-binding effectors. In terms of signalling, the FGFRs also bind to HS and the ternary signalling ligand-receptor complex involves the FGF ligand, HS co-receptor and FGFR. Work in cultured cells indicates that in at least some instances the structure of the polysaccharide can control the formation of signalling complexes independently of ligand binding [54,64] and so the selectivity of an FGF ligand–FGFR pair may differ from that of the individual proteins [65]. Ternary signalling complexes have been found in other families of HS-binding effectors, so this mode of regulation may be more widespread. The divergence of the HS binding properties of FGFs may have been constrained by these impacting on different facets of FGF function and by the specificity code being three-dimensional, rather than linear. The interaction of FGFR with HS and the functional requirements of other families of HS-binding effectors would then provide additional constraints on the divergence of HS binding properties.

## Supplementary Material

Li et al FGF -GAG interactions supplemental

## Data accessibility

The mass spectrometry spectrums supporting this article have been uploaded as part of the electronic supplementary material.
