# Supplementary material for: Heparin binding preference and structures in the fibroblast growth factor family parallel their evolutionary diversification
Source: Open Biol. 2016 Mar 30;6(3):150275. doi: 10.1098/rsob.150275 (PMC4821243; doi:10.1098/rsob.150275)

Supplemental Information

Heparin binding preference and structures in the fibroblast growth factor family parallel their evolutionary diversification

Yong Li[1], Changye Sun[1], Edwin A. Yates[1], Chao Jiang[2], Mark C. Wilkinson[1], David G. Fernig[1]

[1]Department of Biochemistry, Institute of Integrative Biology, Biosciences Building, University of Liverpool, Crown Street, Liverpool L69 7ZB, United Kingdom

[2]School of Pharmaceutical Science, Wenzhou Medical University, Chashan University Park, Wenzhou 325035, China

Corresponding Author:

David G. Fernig, Department of Biochemistry, Biosciences Building, University of Liverpool, Crown Street, Liverpool L69 7ZB, UK. Tel.: +441517954471; Fax: +441517954406; E-mail: dgfernig@liverpool.ac.uk.

Table of contents

Figure S1 Stabilization effect of heparin on FGF10 3

Figure S2 Stabilization effect of heparin on FGF4 4

Figure S3 Stabilization effect of heparin on HT-FGF6 5

Figure S4 Stabilization effect of heparin on FGF17 6

Figure S5 Stabilization effect of heparin on FGF20 7

Figure S6 The average molecular mass per volume unit volume and the differential refractive index of FGF20 8

Table S1 Summary of peptides of FGFs identified by lysine targeted Protect and Label structural proteomics 9

Figure S7 Peptides corresponding to trypsin digested FGF3 10

Figure S8 Peptides corresponding to thermolysin digested FGF3 11

Figure S9 Peptides corresponding to chymotrypsin digested FGF3 12

Figure S10 Peptides corresponding to trypsin digested FGF10 13

Figure S11 Peptides corresponding to chymotrypsin digested FGF10 14

Figure S12 Peptides corresponding to chymotrypsin digested FGF4 15

Figure S13 Peptides corresponding to chymotrypsin digested FGF4 (2) 16

Figure S14 Peptides corresponding to thermolysin digested FGF4 17

Figure S15 Peptides corresponding to trypsin digested HT-FGF6 18

Figure S16 Peptides corresponding to chymotrypsin digested HT-FGF6 19

Figure S17 Peptides corresponding to Glu-C digested FGF17 20

Figure S18 Peptides corresponding to chymotrypsin digested FGF17 21

Figure S19 Peptides corresponding to thermolysin digested FGF17 22

Figure S20 Peptides corresponding to trypsin and Glu-C digested FGF17 23

Figure S21 Peptides corresponding to trypsin digested FGF17 24

Figure S22 Peptides corresponding to chymotrypsin digested FGF20 25

Figure S23 Peptides corresponding to thermolysin digested FGF20 26

Figure S1 Stabilization effect of heparin on FGF10. Differential scanning fluorimetry of 5 μM FGF10 in the presence of various concentrations of heparin. (*a*) melting curve profiles of FGF10 (5 μM) with a range of heparin concentrations (0-100 μM). (*b*) first derivative of the melting curves of FGF10 in (*a*). (*c*) peak of the first derivative of the melting curves from (*b*), which is the melting temperature, Tm (mean of triplicate ± S.E.).

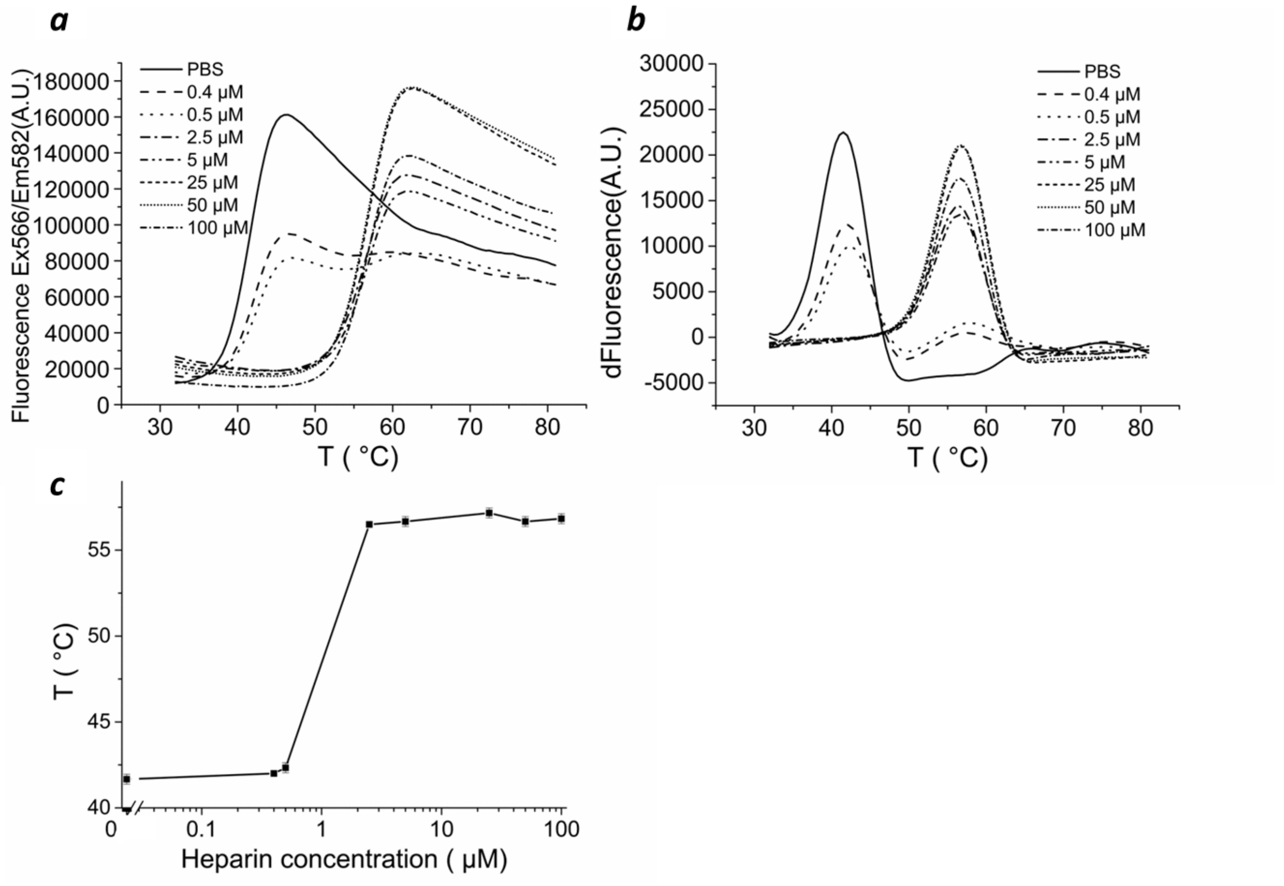

Figure S2 Stabilization effect of heparin on FGF4. Differential scanning fluorimetry of 5 µM FGF4 in the presence of various concentrations of heparin. (*a*) melting curve profiles of FGF4 (5 µM) with a range of heparin concentrations (0-100 µM). (*b*) first derivative of the melting curves of FGF4 in (*a*). (*c*) peak of the first derivative of the melting curves from (*b*), which is the melting temperature, Tm (mean of triplicate ± S.E.).

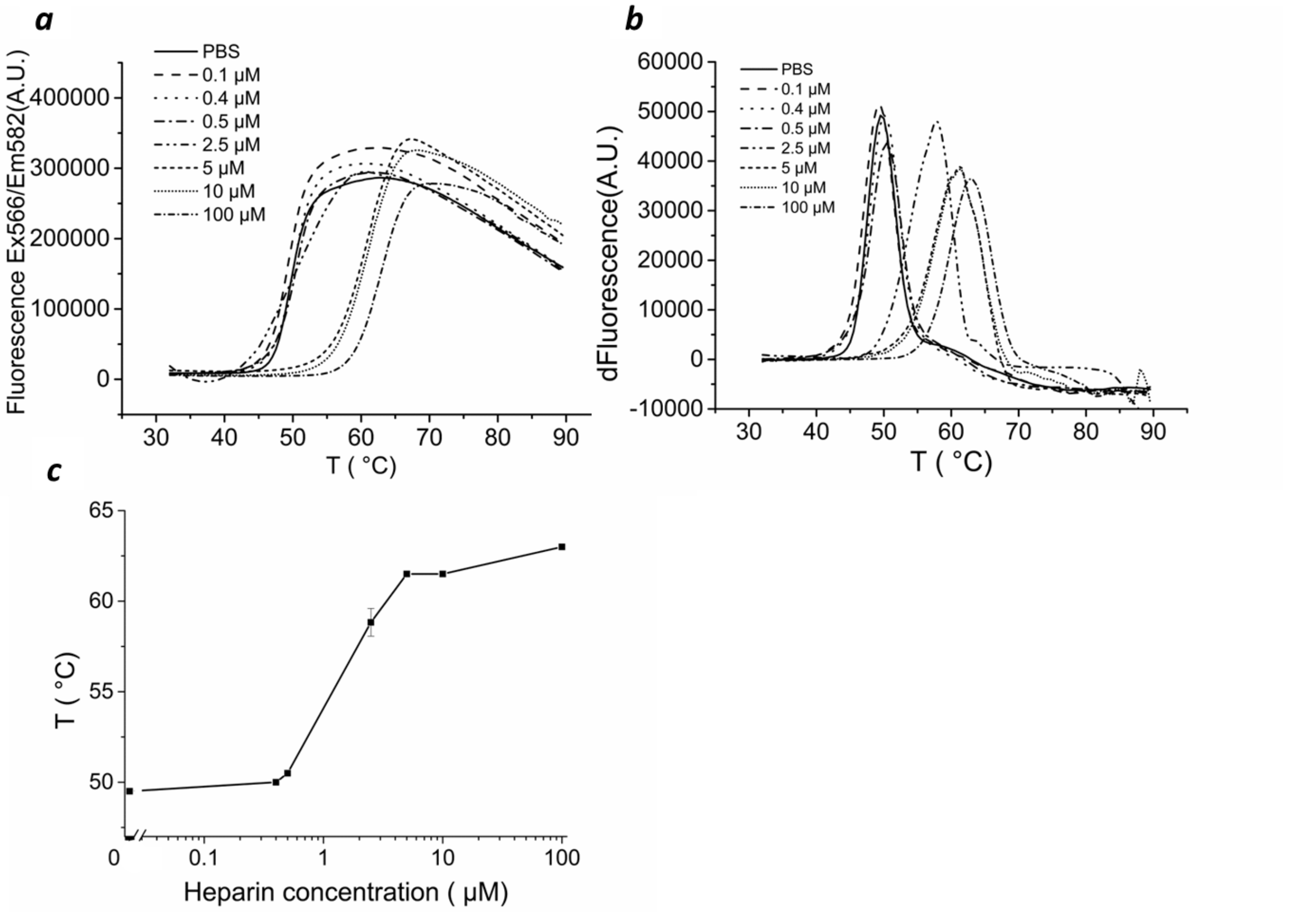

Figure S3 Stabilization effect of heparin on HT-FGF6. Differential scanning fluorimetry of 5 µM HT-FGF6 in the presence of various concentrations of heparin. (*a*) melting curve profiles of HT-FGF6 (5 µM) with a range of heparin concentrations (0-100 µM). (*b*) first derivative of the melting curves of HT-FGF6 in (*a*). (*c*) the melting temperature of Halo protein with and without heparin (2.5 µM and 10 µM). (*d*) peak of the first derivative of the melting curves from (*b*), which is the melting temperature, Tm (mean of triplicate ± S.E.).

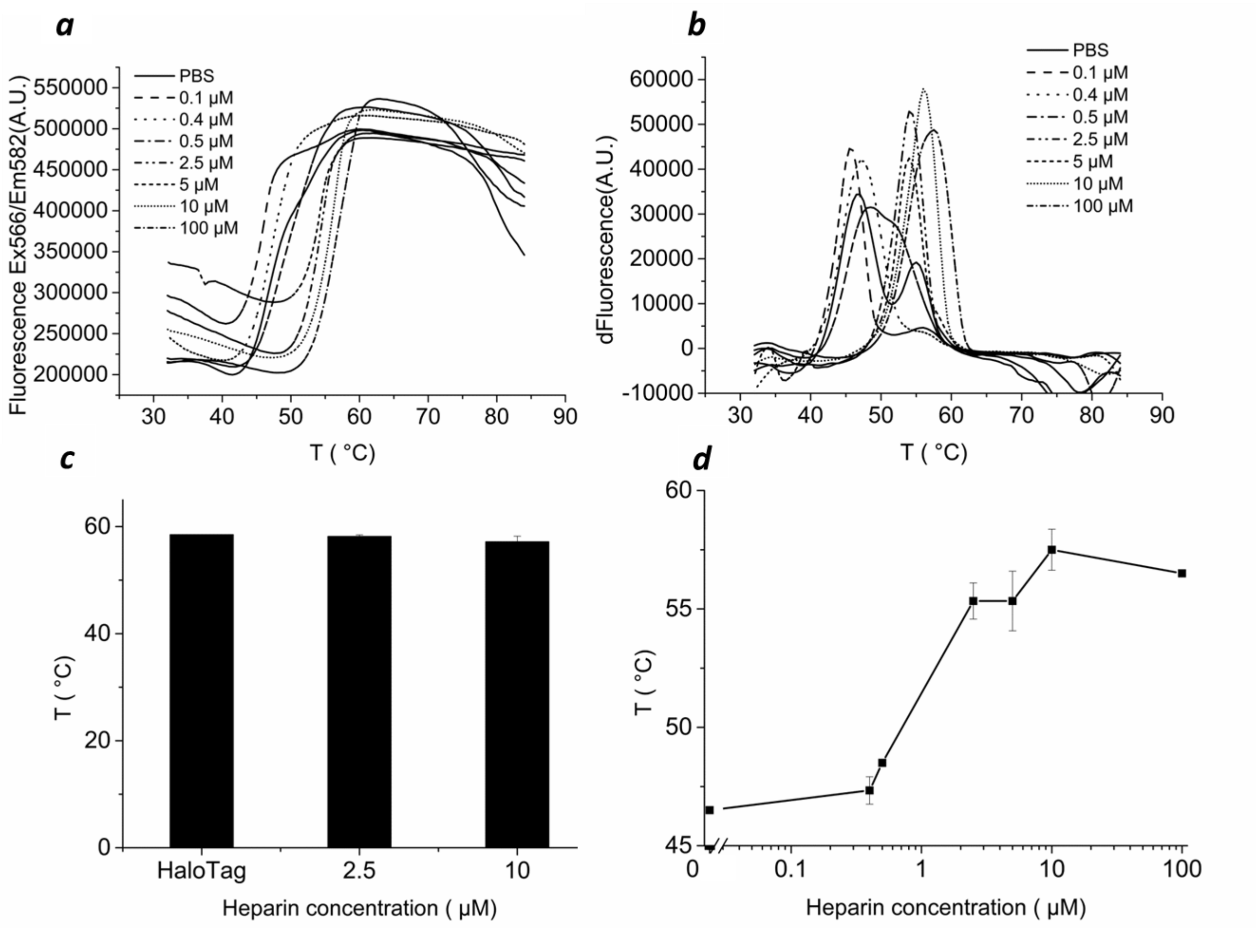

Figure S4 Stabilization effect of heparin on FGF17. Differential scanning fluorimetry of 5 µM FGF17 in the presence of various concentrations of heparin. (*a*) melting curve profiles of FGF17 (5 µM) with a range of heparin concentrations (0-100 µM). (*b*) first derivative of the melting curves of FGF17 in (*a*). (*c*) peak of the first derivative of the melting curves from (*b*), which is the melting temperature, Tm (mean of triplicate ± S.E.).

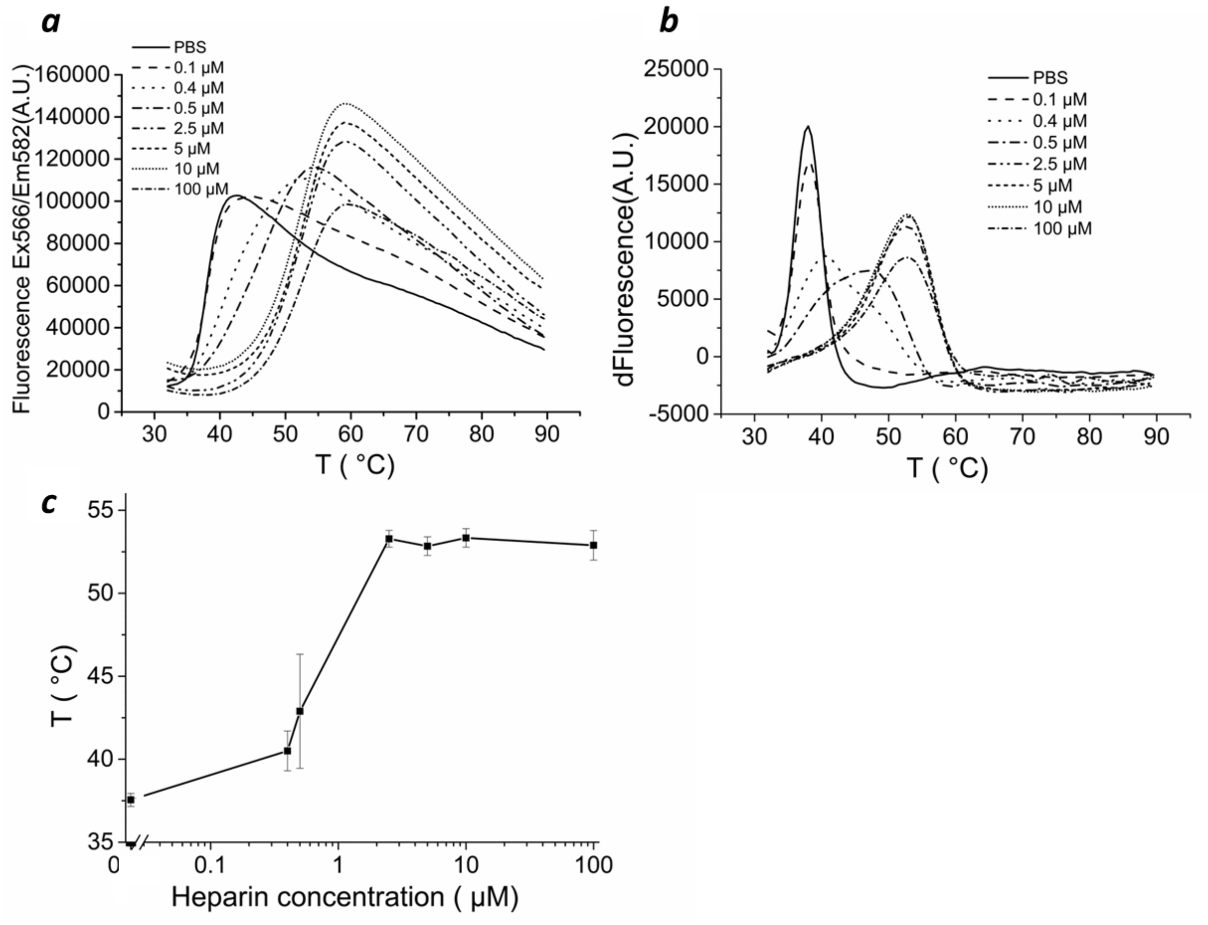

Figure S5 Stabilization effect of heparin on FGF20. Differential scanning fluorimetry of 5 μM FGF20 in the presence of various concentrations of heparin. (*a*) melting curve profiles of FGF20 (5 μM) with a range of heparin concentrations (0-100 μM). (*b*) first derivative of the melting curves of FGF20 in (*a*). (*c*) peak of the first derivative of the melting curves from (*b*), which is the melting temperature, Tm (mean of triplicate ± S.E.).

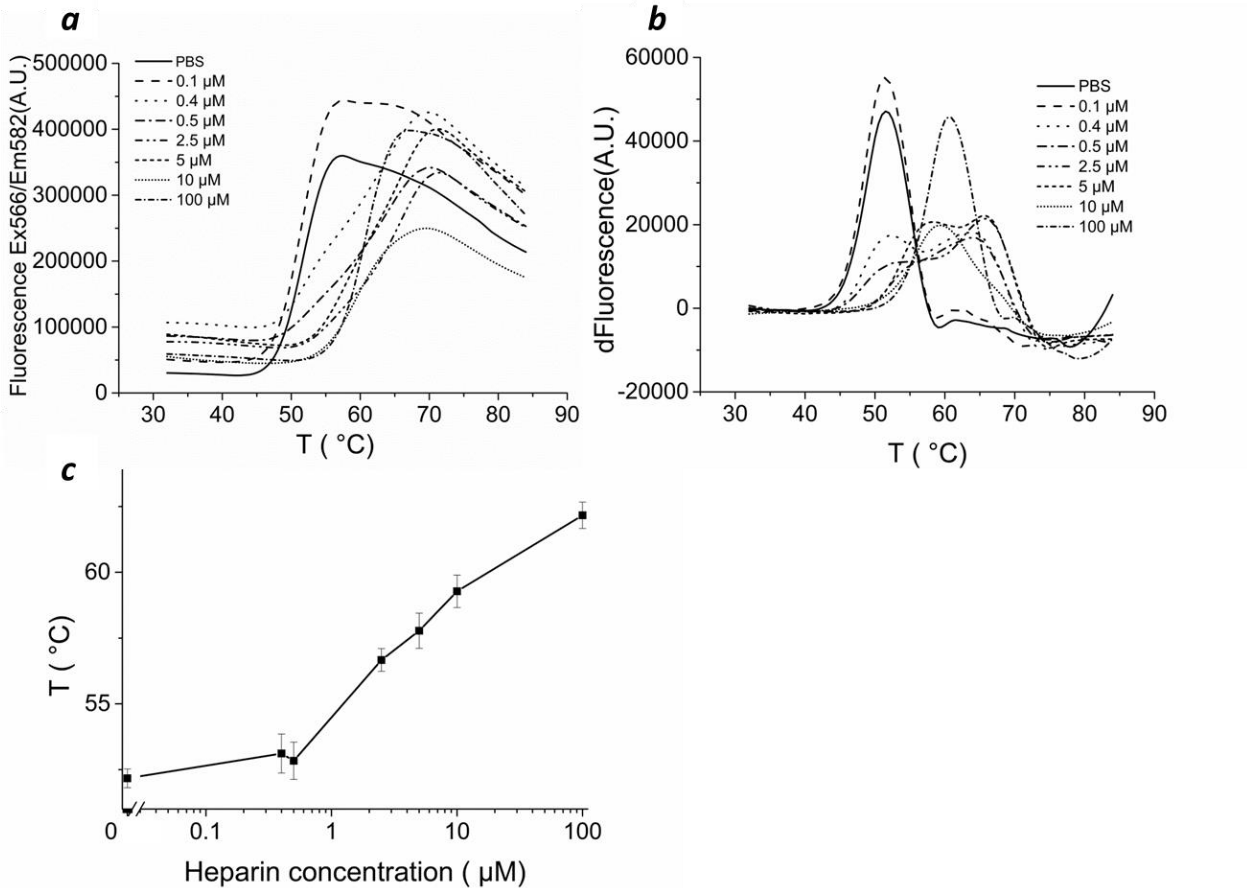

Figure S6 The average molecular mass per volume unit volume and the differential refractive index of FGF20. The red line shows the different reflective index (dRI). The blue line indicates the molecular mass of FGFs.

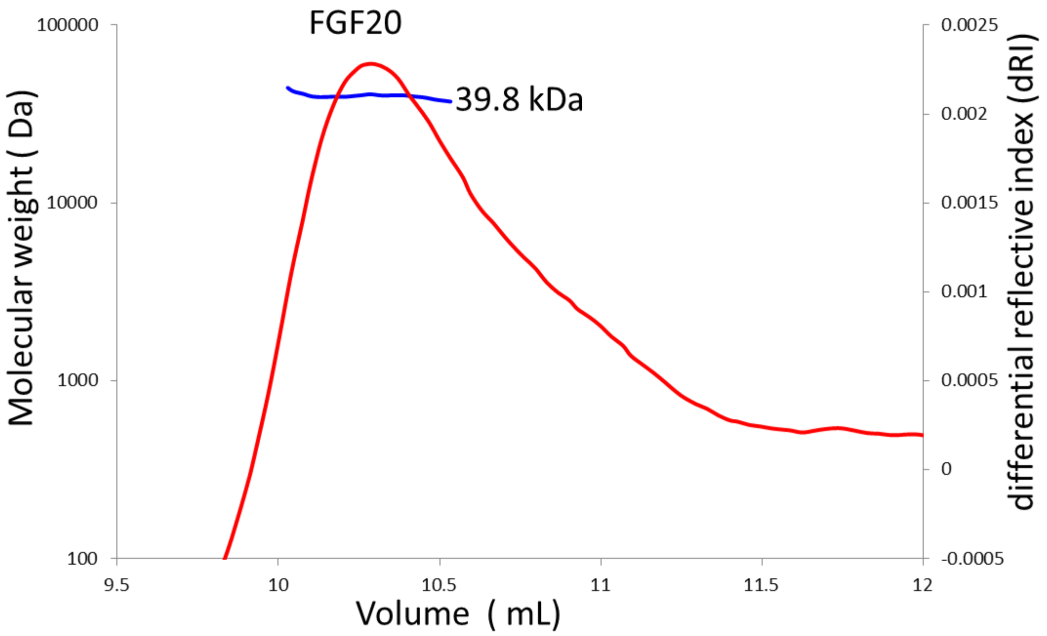

Table S1

Summary of peptides of FGFs identified by lysine targeted Protect and Label structural proteomics

Labelled peptides were identified by MALDI-Q-TOF and analysed by MS-digest from the package ProteinProspector v 5.12.3. A full list of identified peptides is provided in this table. Four proteases used for protein digestion were trypsin (TRY), thermolysin (THE), chymotrypsin (CHY), Glu-c (GLU) and mixture of trypsin and Glu-C (TG).

Peptide		Sequences	Proteases	Residues	HBS	Spectrum
FGF3	1	YC(Carbamidomethyl)ATK(biotin)YHLQ	(THE)	49-57	1	S8
	2	RTQK(biotin)SSLFLPR	(TRY)	171-181	1	S7
FGF10	1	FSFTK(biotin)Y	(CHY)	83-88	1	S11
FGF17	1	AK(biotin)LIVETDTF	(CHY)	84-93	1	S18
	2	AHFIK(biotin)R	(TRY)	172-177	2	S21
	3	EAHFIK(biotin)R	(TG)	171-177	2	S20
	4	LYQGQLPFPNHAEK(biotin)QK(biotin)QFE	(THE)	178-196	2	S19
	5	K(biotin)QK(biotin)QFE	(GLU)	191-196	2	S17
FGF4	1	VAMSSK(biotin)GK(biotin)	(CHY)	137-145	1	S12
	2	IALSKNGKTKKGNRVSPT(3biotin/1acetyl)	(THE)	179-196	1	S13
	3	AGDYLLGIK(biotin)R	(THE)	73-82	3	S13
FGF6	1	QGTYIALSK(biotin)Y	(CHY)	177-186	1	S16
	2	ATPSFQEEC(carbamidomethyl)K(biotin)F	(CHY)	149-156	3	S16

Figure S7 Peptides corresponding to trypsin digested FGF3, (Data summary in Tables 2 and S1).

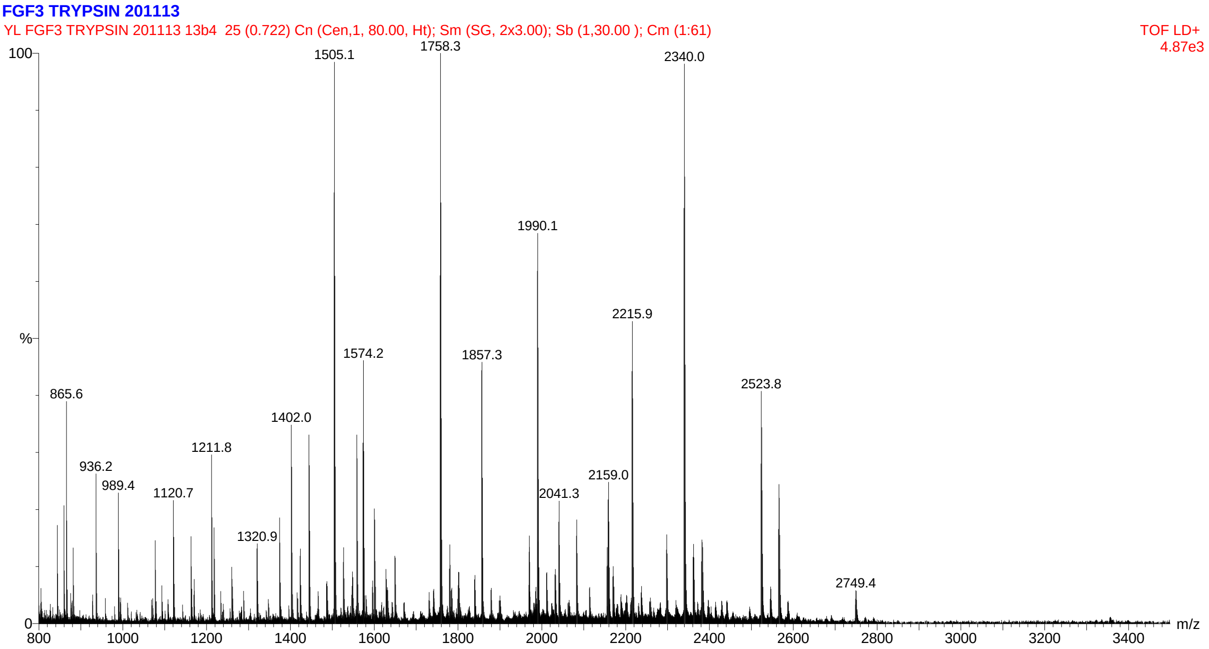

Figure S8 Peptides corresponding to thermolysin digested FGF3, (Data summary in Table 2 and S1).

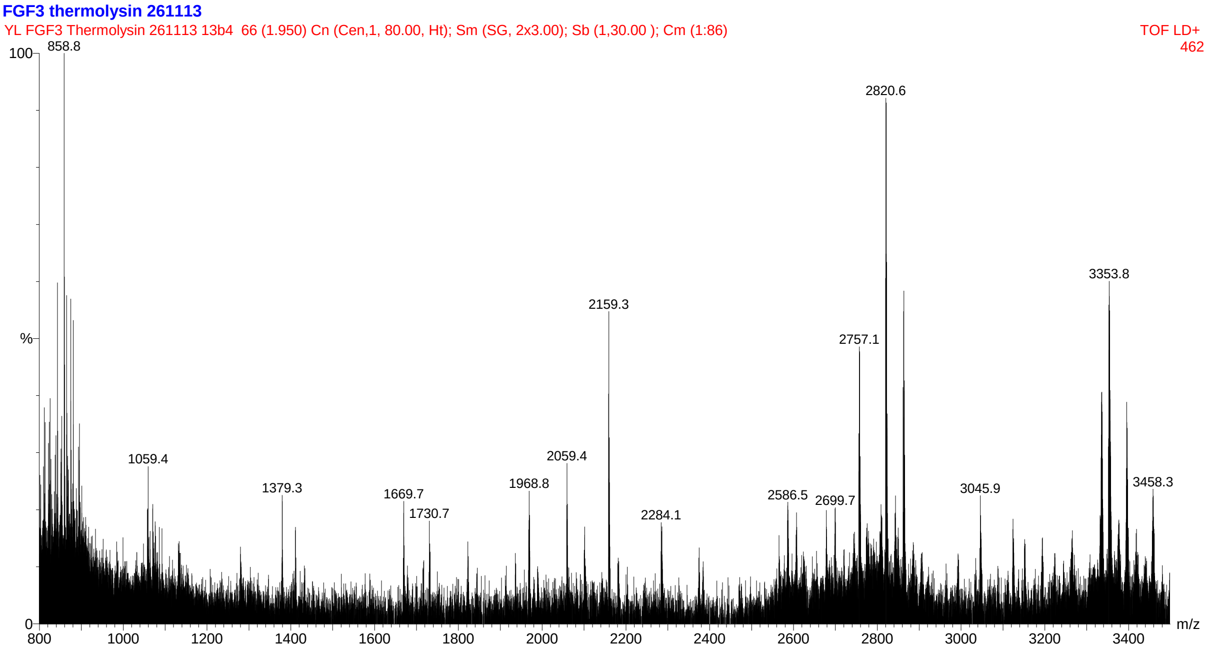

Figure S9 Peptides corresponding to chymotrypsin digested FGF3, (Data summary in Table 2).

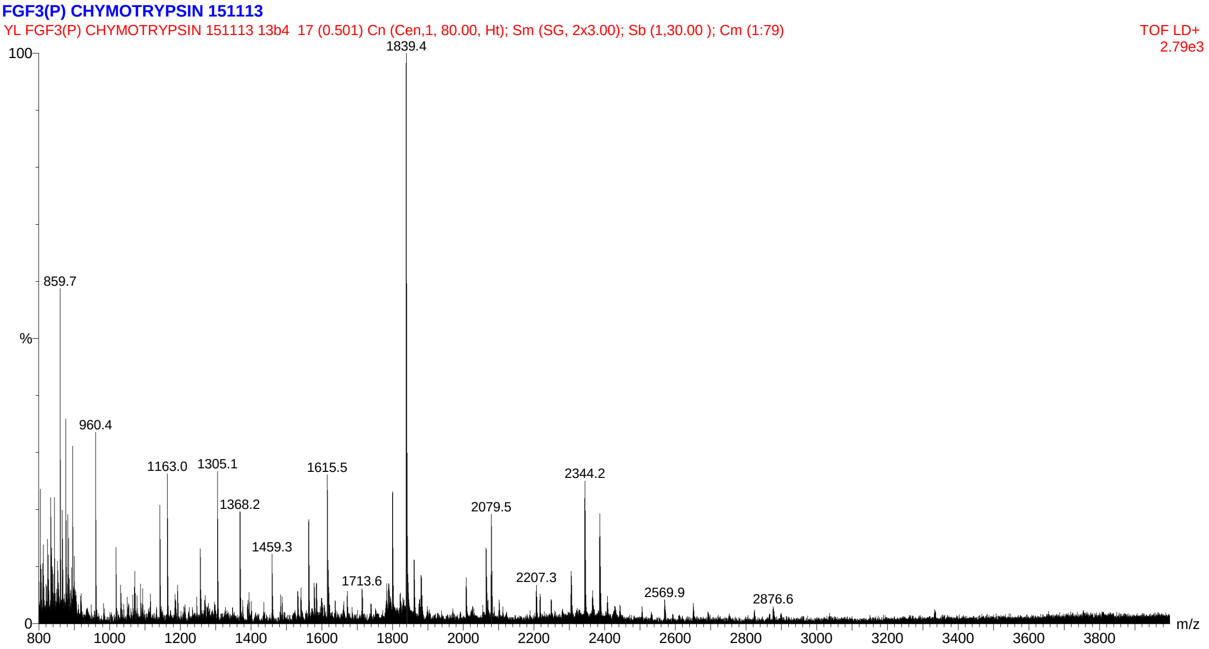

Figure S10 Peptides corresponding to trypsin digested FGF10, (Data summary in Table 2).

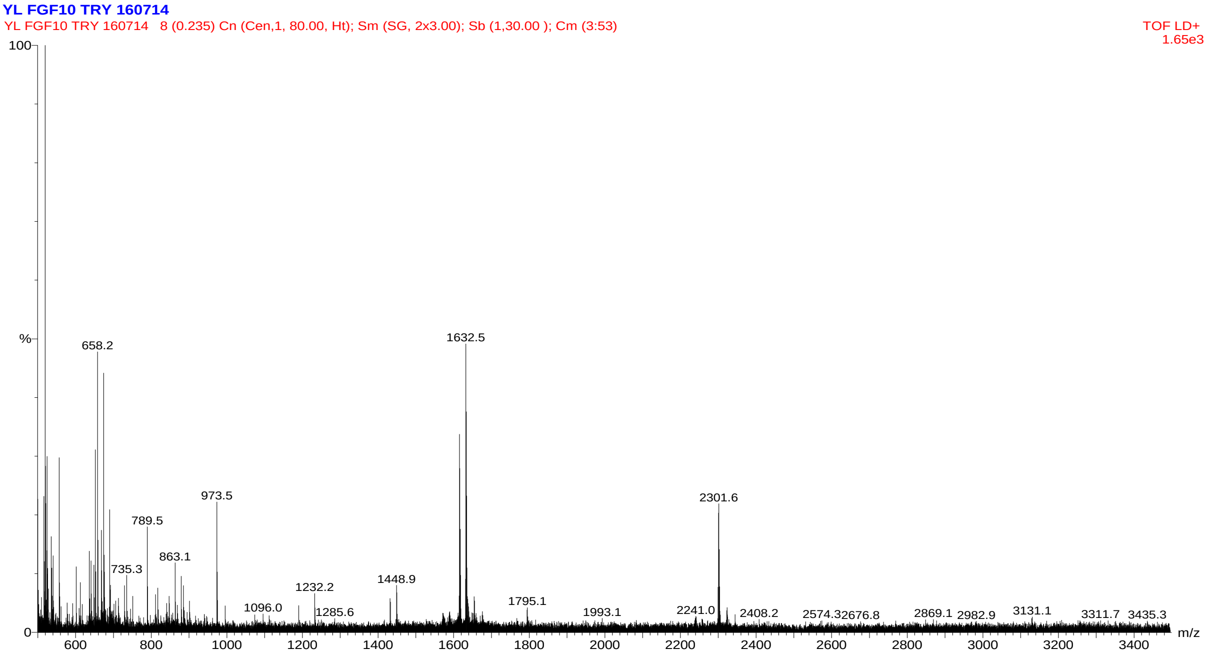

Figure S11 Peptides corresponding to chymotrypsin digested FGF10, (Data summary in Table 2 and S1).

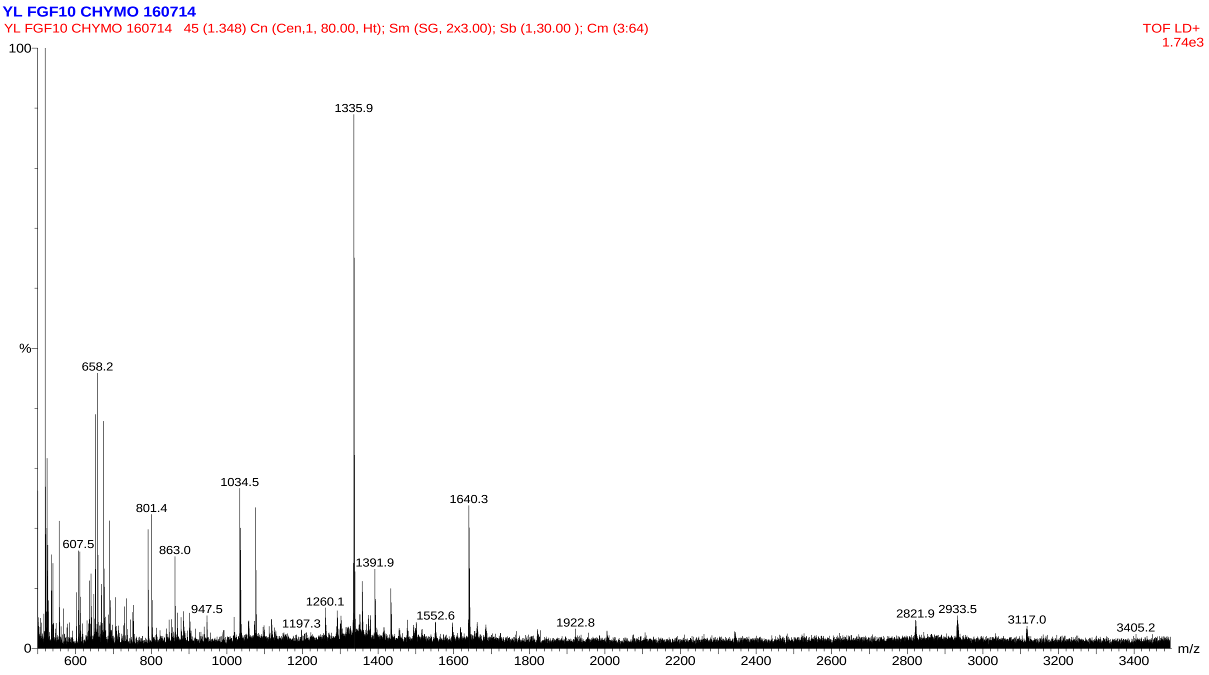

Figure S12 Peptides corresponding to chymotrypsin digested FGF4, (Data summary in Table 3 and S1). Due to variability in the digestion of FGF4, two spectra are shown (Figures S12 and S13).

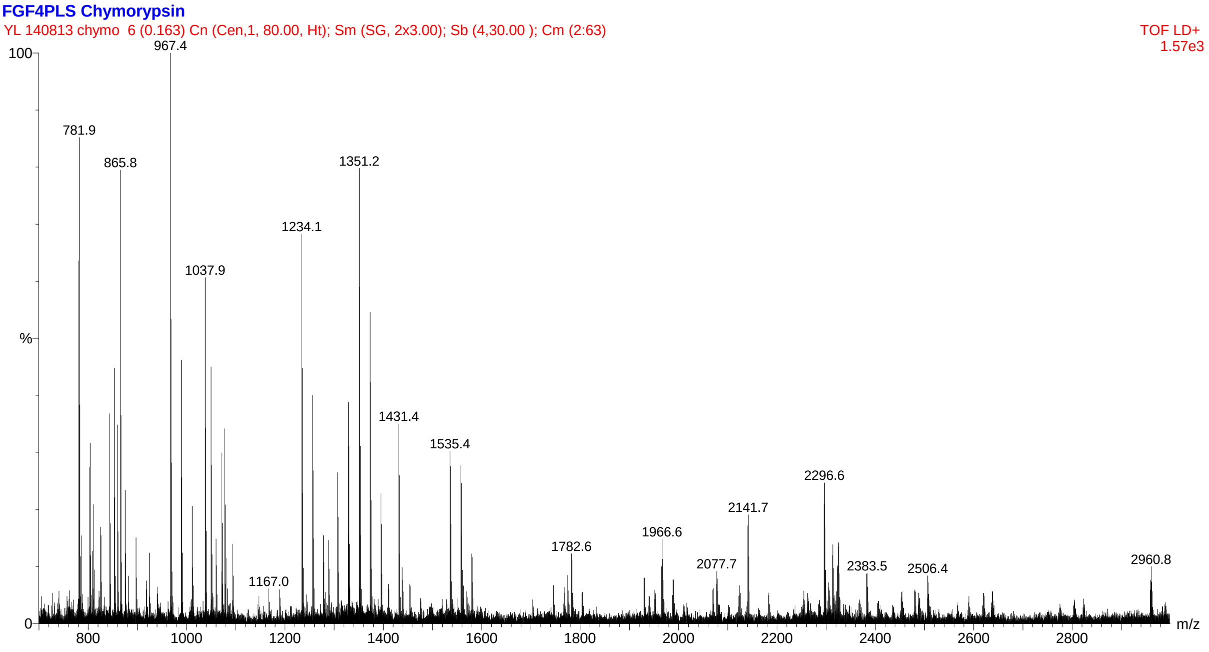

Figure S13 Peptides corresponding to chymotrypsin digested FGF4, (Data summary in Table 3 and S1). Due to variability in the digestion of FGF4, two spectra are shown (Figures S12 and S13).

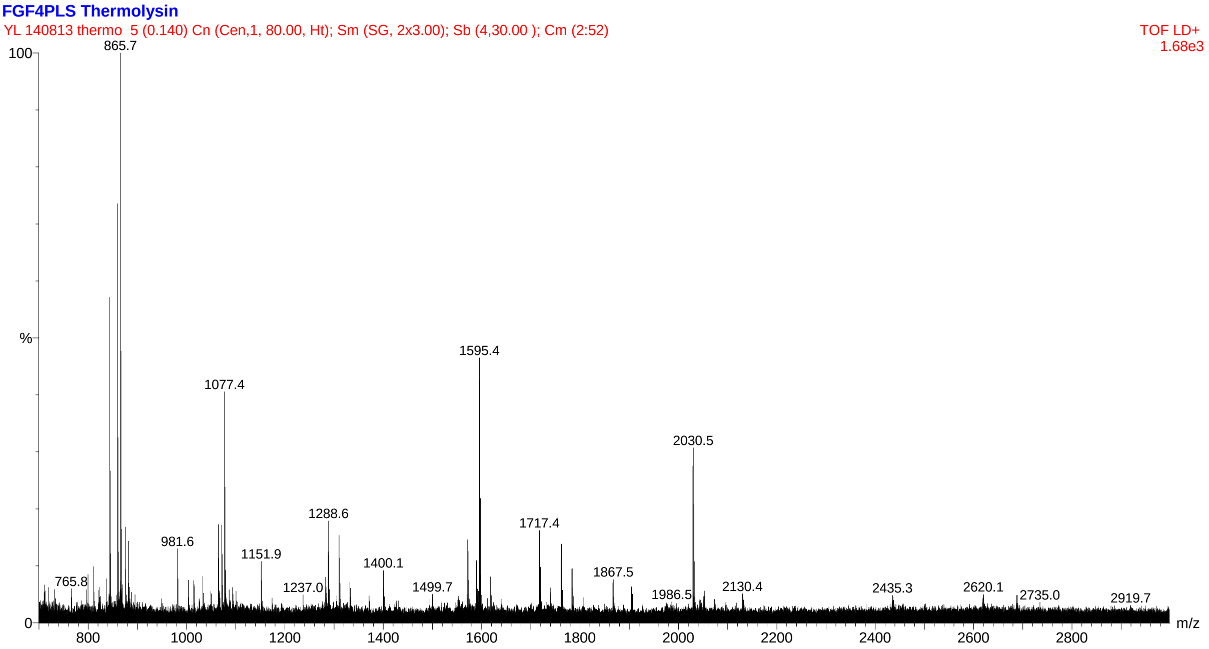

Figure S14 Peptides corresponding to thermolysin digested FGF4 (2), (Data summary in Table 3).

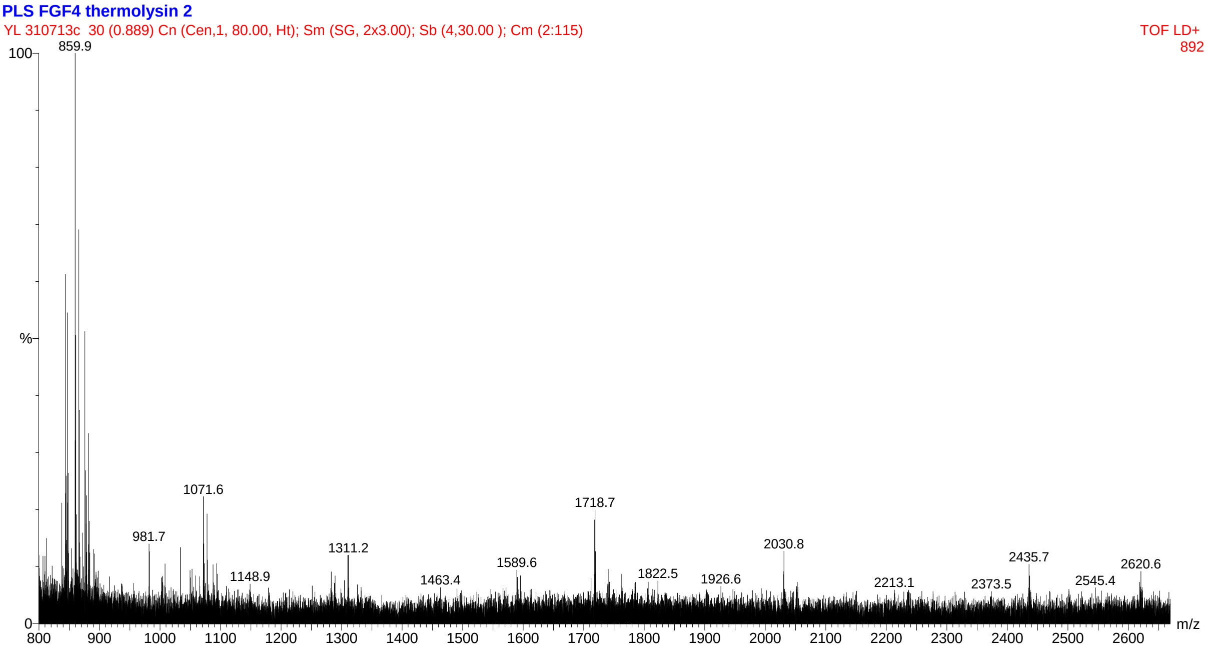

Figure S15 Peptides corresponding to trypsin digested HT-FGF6, (Data summary in Table 3).

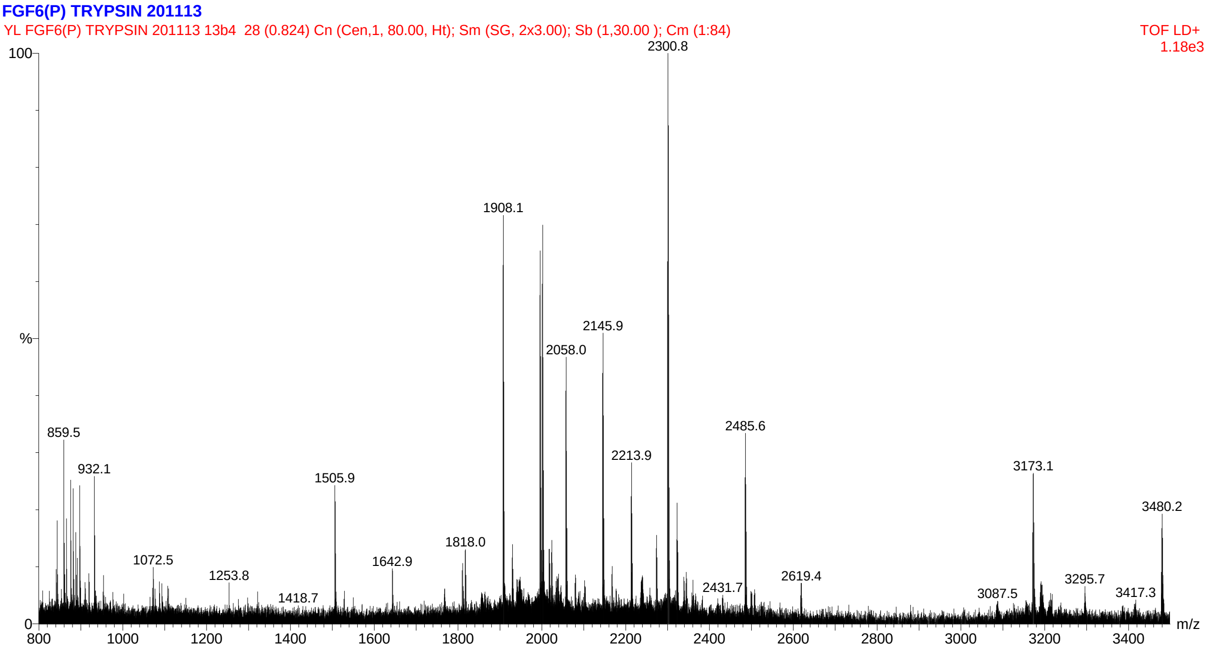

Figure S16 Peptides corresponding to chymotrypsin digested HT-FGF6, (Data summary in Table 3 and S1).

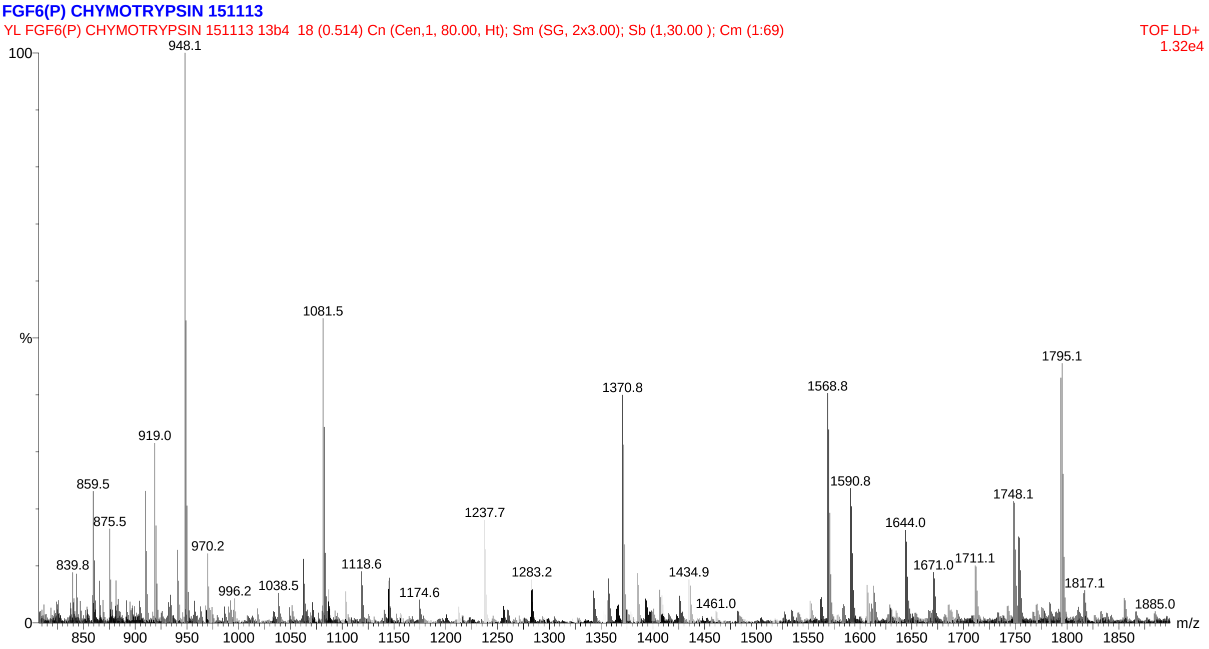

Figure S17 Peptides corresponding to Glu-C digested FGF17, (Data summary in Table 4 and S1).

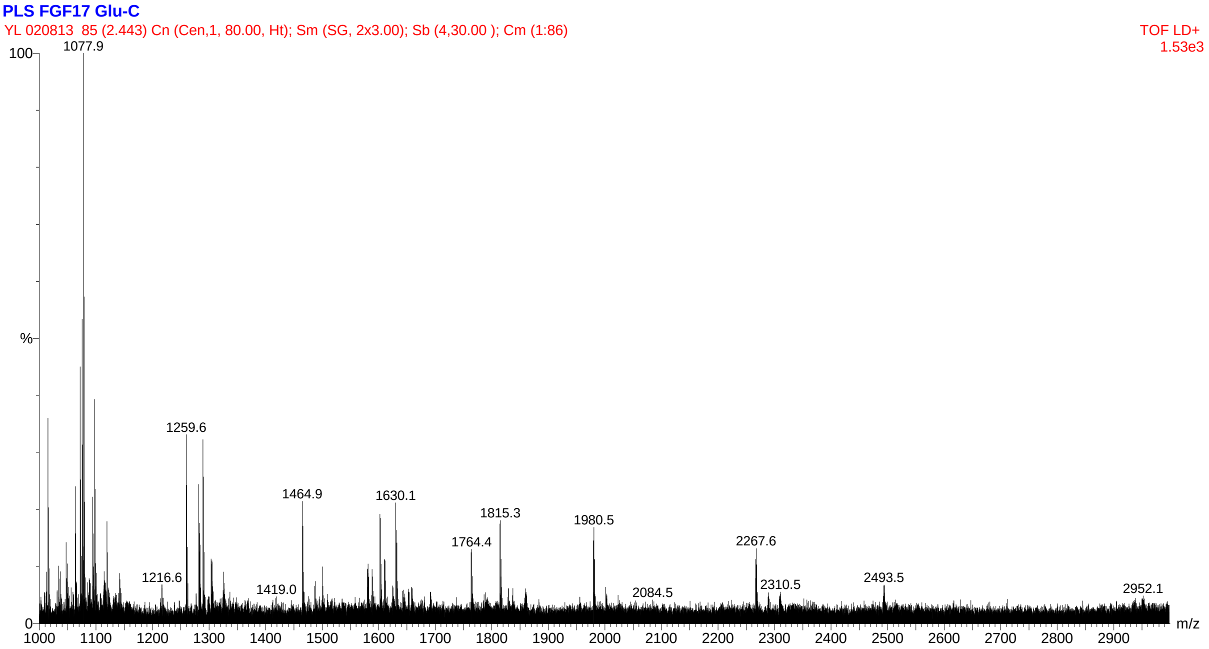

Figure S18 Peptides corresponding to chymotrypsin digested FGF17, (Data summary in Table 4 and S1).

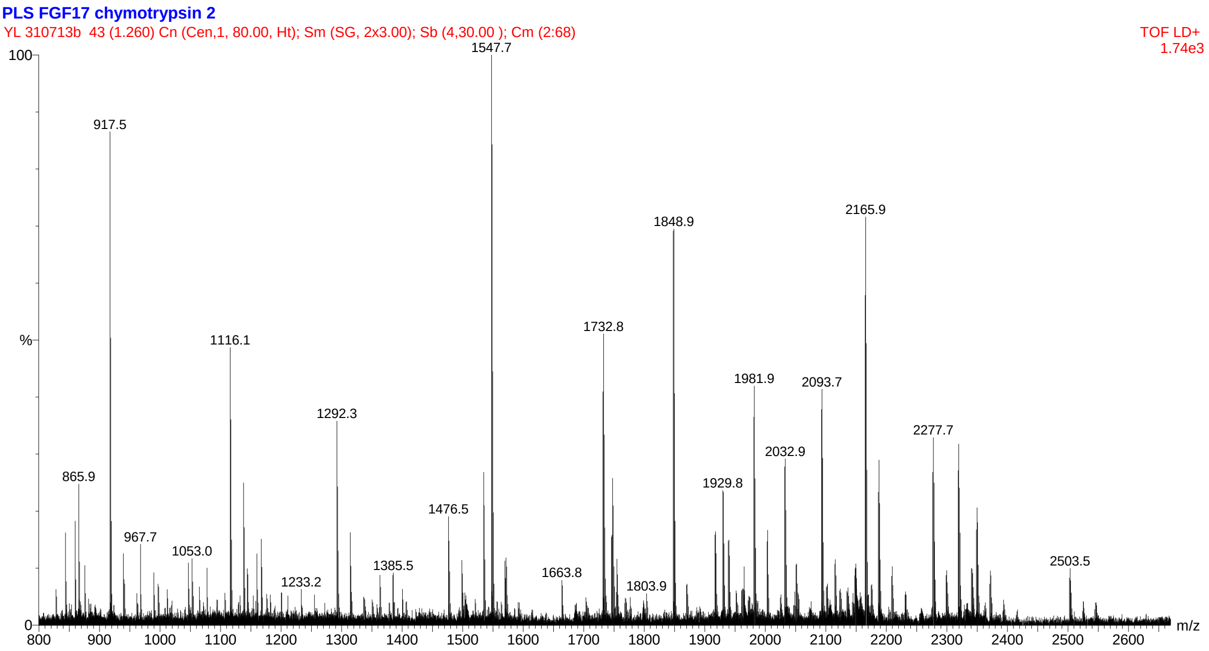

Figure S19 Peptides corresponding to thermolysin digested FGF17, (Data summary in Table 4 and S1).

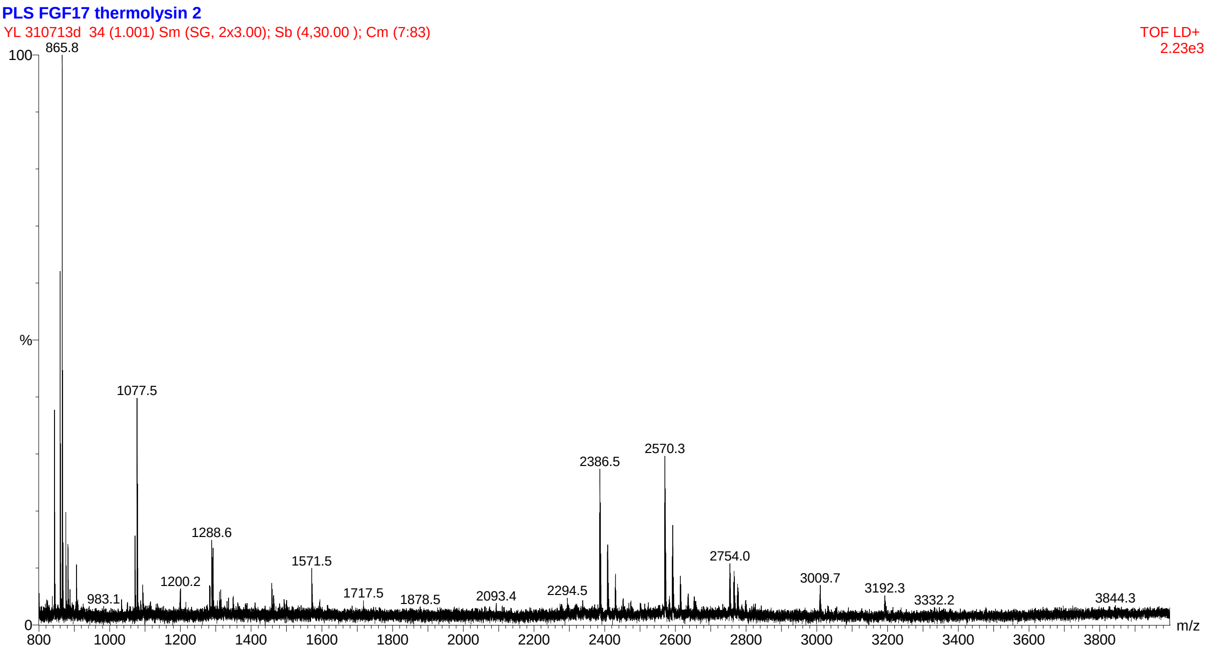

Figure S20 Peptides corresponding to trypsin and Glu-C digested FGF17, (Data summary in Table S1).

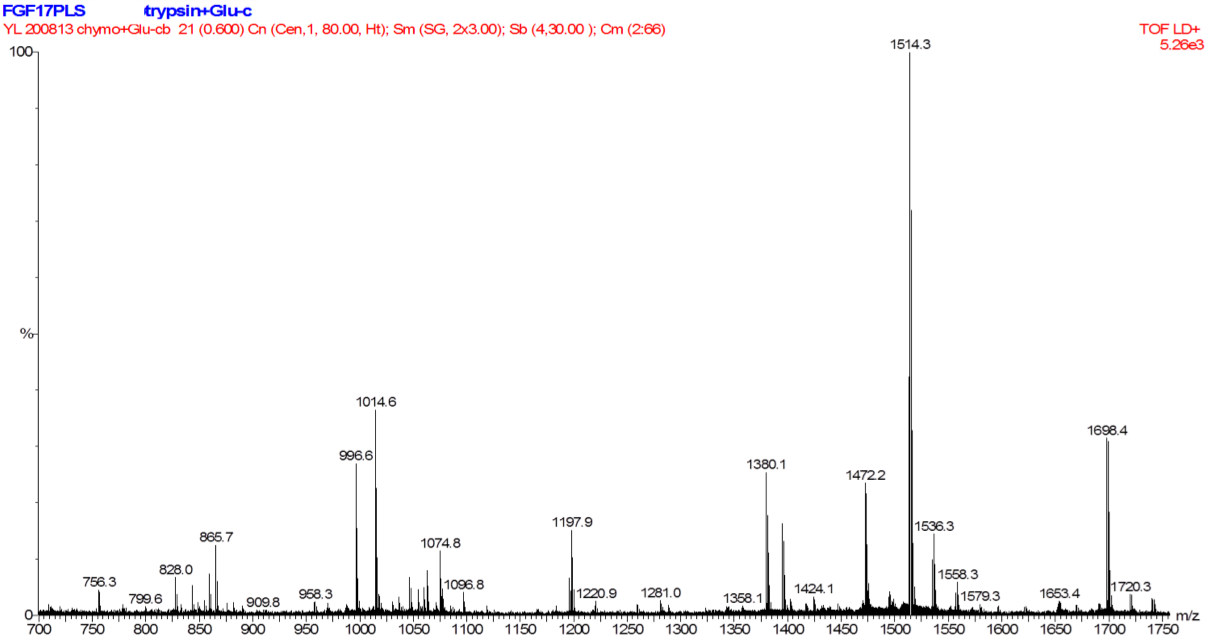

Figure S21 Peptides corresponding to trypsin digested FGF17, (Data summary in Table S1).

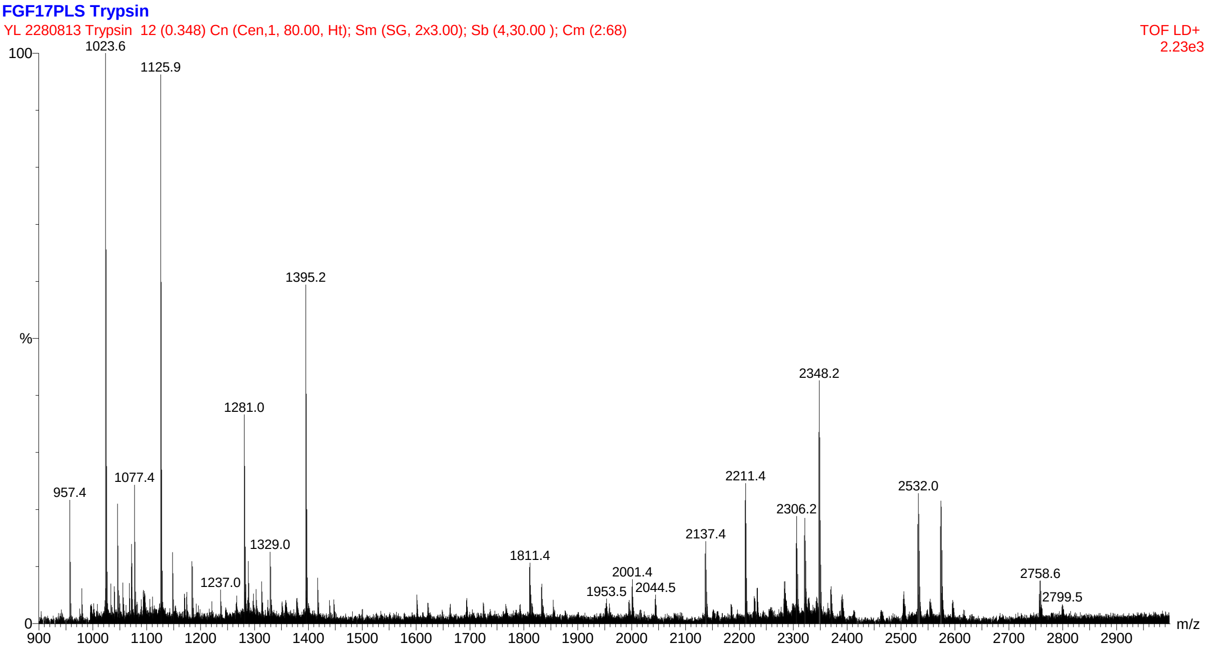

Figure S22 Peptides corresponding to chymotrypsin digested FGF20, (Data summary in Table 5).

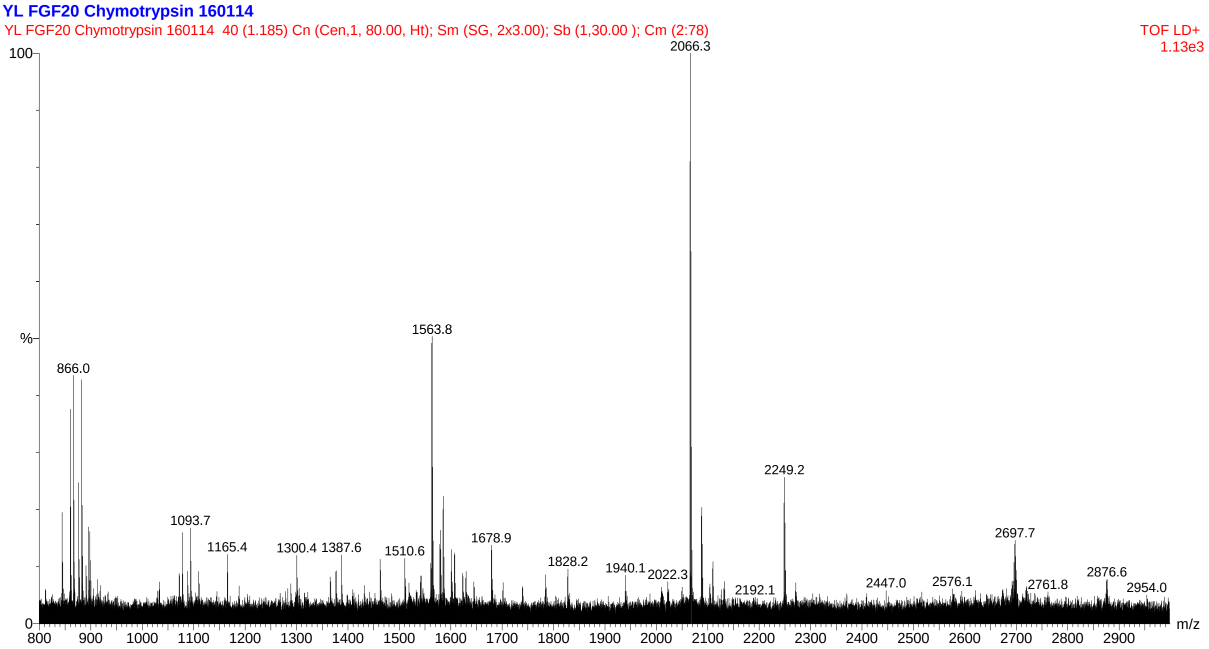

Figure S23 Peptides corresponding to thermolysin digested FGF20, (Data summary in Table 5).

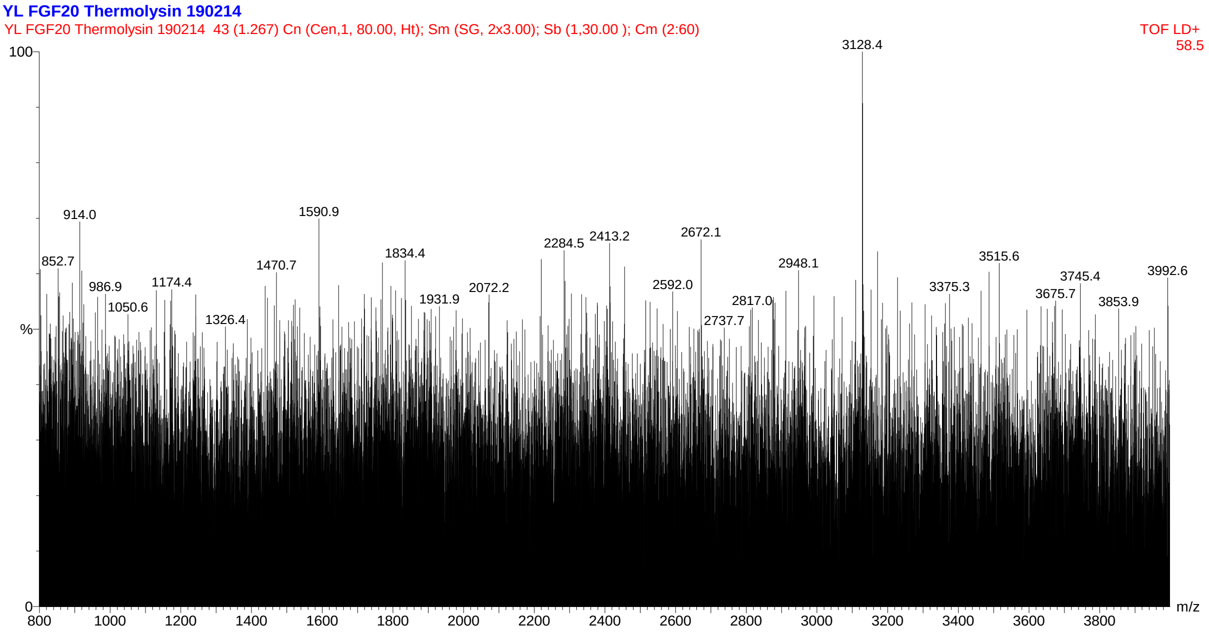

Supplement: Li et al FGF -GAG interactions supplemental [file rsob150275supp1.pdf]
